# Development of ^177^Lu-scFvD2B as a Potential Immunotheranostic Agent for Tumors Overexpressing the Prostate Specific Membrane Antigen

**DOI:** 10.1038/s41598-020-66285-2

**Published:** 2020-06-09

**Authors:** Debora Carpanese, Guillermina Ferro-Flores, Blanca Ocampo-Garcia, Clara Santos-Cuevas, Nicola Salvarese, Mariangela Figini, Giulio Fracasso, Laura De Nardo, Cristina Bolzati, Antonio Rosato, Laura Meléndez-Alafort

**Affiliations:** 1 Istituto Oncologico Veneto IOV-IRCCS, Padua, Italy; 2 Laboratorio Nacional de Investigación y Desarrollo de Radiofármacos-CONACyT, Instituto Nacional de Investigaciones Nucleares, Estado de México, Mexico; 3 Institute of Condensed Matter Chemistry and Energy Technologies, National Research Council, Padua, Italy; 4 Biomarker Unit, Dipartimento di Ricerca Applicata e Sviluppo Tecnologico, Fondazione IRCCS Istituto Nazionale dei Tumori, Milan, Italy; 5 Department of Medicine, University of Verona, Verona, Italy; 6 Department of Physics and Astronomy, University of Padua, Padua, Italy; 7 Department of Surgery, Oncology, and Gastroenterology, University of Padua, Padua, Italy

**Keywords:** Target identification, Tumour biomarkers, Preclinical research

## Abstract

The clinical translation of theranostic ^177^Lu-radiopharmaceuticals based on inhibitors of the prostate-specific membrane antigen (PSMA) has demonstrated positive clinical responses in patients with advanced prostate cancer (PCa). However, challenges still remain, particularly regarding their pharmacokinetic and dosimetric properties. We developed a potential PSMA-immunotheranostic agent by conjugation of a single-chain variable fragment of the IgGD2B antibody (scFvD2B) to DOTA, to obtain a ^177^Lu-labelled agent with a better pharmacokinetic profile than those previously reported. The labelled conjugated ^177^Lu-scFvD2B was obtained in high yield and stability. *In vitro*, ^177^Lu-scFvD2B disclosed a higher binding and internalization in LNCaP (PSMA-positive) compared to PC3 (negative control) human PCa cells. *In vivo* studies in healthy nude mice revealed that ^177^Lu-scFvD2B present a favorable biokinetic profile, characterized by a rapid clearance from non-target tissues and minimal liver accumulation, but a slow wash-out from kidneys. Micro-SPECT/CT imaging of mice bearing pulmonary microtumors evidenced a slow uptake by LNCaP tumors, which steadily rose up to a maximum value of 3.6 SUV at 192 h. This high and prolonged tumor uptake suggests that ^177^Lu-scFvD2B has great potential in delivering ablative radiation doses to PSMA-expressing tumors, and warrants further studies to evaluate its preclinical therapeutic efficacy.

## Introduction

### Background

Prostate cancer (PCa) is the second leading cause of cancer deaths for adult men in the Western world. Although radical prostatectomy and local radiotherapy are largely successful for patients with localized cancer, available treatments for metastatic PCa have demonstrated weak curative efficacy^1^. Consequently, new tools to improve the detection of recurrent PCa, and particularly to identify and treat distant metastases, are imperatively needed.

The prostate-specific membrane antigen (PSMA) is one of the most promising targets for the development of PCa theranostic agents. PSMA is overexpressed in 95% of prostate cancers and its expression levels progressively increase in high-grade tumors and in metastatic disease, up to 1,000 times more than in normal cells^2^. Among the several PSMA-targeting molecules that have been developed, the radiolabeled Glu-ureido-based PSMA inhibitors are gaining much interest due to their high uptake by PSMA-positive cancer cells, and low background and excellent contrast in cancer imaging, even in small metastases^3,4^. Theranostic agents such as ^177^Lu-PSMA-617, ^177^Lu-PSMA-I&T and ^177^Lu-iPSMA have demonstrated their ability to efficiently target PSMA-expressing tumors, with the consequent decrease of serum PSA levels in 30–60% of PCa patients^5,6^. However, the individual dosimetry calculation disclosed high absorbed doses in the lacrimal and salivary glands due to the high retention of ^177^Lu-PSMA-ligands^7^. As consequence, most ^177^Lu-labeled PSMA inhibitors are currently employed only as compassionate treatment for patients with end-stage metastatic castration-resistant PCa^8^.

Antibody-based constructs represent another strategy that has been pursued in order to develop PSMA-imaging or theranostic agents. To date, only the murine monoclonal antibody (mAb) ^111^In-capromab pendetide (ProstaScint®) has been approved by the Food and Drug Administration as a diagnostic agent for PCa. However, ProstaScint® binding sites are exposed only after apoptosis or necrosis processes^9^. Smith-Jones *et. al*. developed the humanized J591 mAb, which recognizes and binds to an extracellular epitope of PSMA with high affinity. Nonetheless, as a whole antibody it exhibits low tumor targeting with a maximum uptake at 6 days post-injection, and delayed clearance from non-target tissues^9^; these issues limit the use of J591 as a theranostic agent^10^. Recently, a new mAb against human PSMA (IgGD2B) was developed using conventional hybridoma technology^11^. ^111^In-IgGD2B has a higher tumor uptake than ProstaScint, but presents the same limitations due to its molecular size^12^. To circumvent this problem, single-chain variable fragments (scFv) of the D2B antibody have been produced^13^. Optical *in vivo* mouse imaging of fluorophore-labeled scFv (scFvD2B) evidenced high specificity and rapid accumulation in PSMA-positive tumors, with no apparent background^13^. Subsequently, recombinant ^111^In-NOTA-scFvD2B displayed some kidney uptake that was significantly reduced when scFvD2B was radiolabeled with I-131^14^. A GMP-grade ^123^I-labelled scFvD2B showed improved antigen-positive tumor uptake with a shorter circulatory half-life, but also an increased uptake in non-target tissues, such as the stomach and thyroid gland, due to the release of I-123 by a process of *in vivo* ^123^I-scFvD2B dehalogenation^15^.

Radiolabelled-antibodies with higher stability can be obtained using metal chelating agents, due to the significant reduction of the radioisotope release^16^. DOTA is one of the primary used chelators for radio-metals chemistry and has proved to be an excellent chelator for Lutetium. Actually, Lu-DOTA complexes show remarkable kinetic and thermodynamic stability, with no significant *in vivo* metal release even after long circulation times^17^.

In this study, scFvD2B was conjugated to DOTA and labeled with ^177^Lu (^177^Lu-scFvD2B) to assess stability, immunoreactivity, binding and internalization properties using PSMA-expressing cells. Additionally, biodistribution studies were carried out in healthy and LNCaP tumor-bearing mice to establish ^177^Lu-scFvD2B pharmacokinetic profile, and to assess its potential as an immunotheranostic agent.

## Methods

### Cell lines

The human prostate cancer LNCaP and androgen-independent bone metastasis PC-3 cell lines were obtained from the American Type Culture Collection (ATCC). The cell subline PC-3-PIP, modified to express high levels of PSMA, was kindly provided by Dr W. Heston (Cleveland, USA).

### Synthesis and characterization of the DOTA-scFvD2B conjugate

All chemicals were purchased from Sigma-Aldrich unless otherwise specified. DOTA (S-2-(4-benzyl-isothiocyanate)−1,4,7,10-tetra-azacyclododecane tetraacetic-acid) was purchased from Macrocyclics.

ScFvD2B (MW 27 kDa) was produced in an eukaryotic system (ExcellGene) and purified on protein L-sepharose column (GE Healthcare) as previously described^13,15^. To synthesize the DOTA-scFvD2B conjugate, a concentrated solution of scFvD2B (10 mg/mL) in 0.2 M sodium carbonate buffer (pH 9.5) was incubated with p-SCN-Bz-DOTA at 37 °C using 1:2, 1:3 1:4 and 1:5 scFv:DOTA molar ratios. The coupling reaction was quenched by adjusting the pH to 7.0 with 0.25 M ammonium acetate buffer, pH 5.5^18^. In order to remove the DOTA excess, the conjugate was washed with 0.25 M ammonium acetate (pH 7.0), using a Vivaspin^®^ centrifugal concentrator (MWCO 5 kDa; Sartorius). Matrix-assisted laser desorption ionization mass spectrosmetry (MALDI-MS) measurements were performed on a REFLEX 4800 Plus MALDI TOF/TOF instrument (AB Sciex) to determine the number of DOTA per each scFvD2B molecule. Desalted solutions of scFvD2B and DOTA-scFvD2B were diluted to a volume ratio of 1:1 in sinapinic acid solution (10 mg/mL in 50:50 acetonitrile/water). Samples with a final concentration of 5 mg/mL were deposited on a metal MALDI target plate and analyzed. The average number of DOTA per scFvD2B was estimated dividing the mass difference between conjugated and unconjugated scFvD2B by the mass of DOTA (551 Da).

The affinity constant value (K_d_) of the DOTA-scFvD2B conjugate was determined by flow cytometry using a BD FACSCanto II cytometer (Becton and Dickinson). PC-3-PIP and PC-3 cells were re-suspended in cold phosphate-buffered saline (PBS) solution with 0.2% of bovine serum albumin and serial dilutions of the samples were added. After a 1-hour incubation period in ice, cells were washed and stained with saturating amounts of Protein-L Biotin (Life Technologies) in PBS solution over ice for 30 min. Then, cells were washed again and stained with saturating amounts of fluorescein isothiocyanate labeled Avidin (Vector Laboratories). Cell-associated fluorescence was measured by flow cytometry; the percentage of positive cells and the mean fluorescence intensity values were considered. For each sample, under both saturating conditions, the mean fluorescence intensity value was proportional to the number of PSMA sites; therefore, data was expressed as percent saturation of the total stainable PSMA sites. Blocking experiments were also performed on PSMA expressing LNCaP cells, using Fluorescein-5-isothiocyanate (FITC) labeled mAb D2B and increasing concentrations of scFvD2B or its DOTA derivative as described by Parker *et al*.^19^.

### Radiolabeling of PSMA target agents

To establish the minimal amount of DOTA-scFvD2B necessary to obtain ^177^Lu-DOTA-scFvD2B (^177^Lu-scFvD2B) with a high radiochemical yield, 0.5 mL of 0.25 M sodium acetate buffer (pH 7.0), containing different concentrations of DOTA-scFvD2B (15, 30, 60 and 75 nmol), were incubated with 15 µL (from 100 to 370 MBq) of no-carrier-added ^177^LuCl_3_ (ITG) for 2 h at 37 °C. The radiochemical yield of ^177^Lu-scFvD2B was assessed at 1 and 2 h by high performance liquid chromatography (HPLC). Analyses were carried out using a Waters instrument running Empower software with both radioactivity and UV-photodiode array in-line detectors, using a size-exclusion Zorbax GF-250 column (4.59 μm, 9 × 250 mm) from Agilent, and 0.1 M phosphate buffer, pH 7.0, at a 1 mL/min flow rate as mobile phase.

For comparative purposes, ^177^Lu-iPSMA was obtained from the lyophilized kit (ININ-Mexico)^20^, while ^177^Lu-PSMA-617 from DOTA-PSMA-617 purchased from ABX, and used as positive controls. Radiolabeled reactions were carried out, by addition of 0.5 mL (100 MBq) of ^177^LuCl_3_ solution at pH 5 to the DOTA-conjugates and incubation for 30 min at 95 °C in a dry bath as described elsewhere^21^. The radiochemical yield was evaluated by instant thin layer chromatography in glass microfiber paper impregnated with a silica gel (ITLC-SG), using 10-cm strips of Whatman paper and a solution of NaCl 0.9% in 0.02 M HCl as mobile phase. In this system, the free ^177^Lu travels to the front (Rf = 9–10) and the ^177^Lu-PSMA conjugates remain close to the origin (Rf = 1-2).

Stability studies were performed by diluting 0.2 nmol of ^177^Lu-scFvD2B in 100 µL of phosphate buffer (0.1 M; pH 7.0) or human serum, and incubated for 192 h at 37 °C. ^177^Lu-scFvD2B stability was assessed at different time points using radio-HPLC method, as previously reported.

### Cell binding and internalization

To assess the ^177^Lu-radiotracers cell uptake, a solution containing 1 × 10^5^ LNCaP or PC-3 cells diluted in 1 mL of fresh medium was added to a glass tube and incubated with 10 µL (2 MBq and 120 pmol) of ^177^Lu-labeled scFvD2B, iPSMA or PSMA-617 at 37 °C for 1 h (in triplicate). Subsequently, cells were washed twice with PBS solution and counted in a gamma-counter. The internalized fraction of ^177^Lu-PSMA conjugates was calculated after elimination of the membrane-bound radiotracer, by incubating cells with 1 mL of glycine buffer (pH 2.8) for 2 min, washing them with PBS and counting the cell pellet. Total uptake and internalization were expressed as moles of scFvD2B (pmol) in the cell pellet (10^5^ cells) with regard to the total amount (pmol) of the scFvD2B added.

### *In vivo* studies

Animal studies were performed according to national and international ethical regulations, for the handling of laboratory animals (Official Mexican Norm 062-ZOO-1999). This research was approved by the CICUAL-ININ Ethics Committee (Internal Committee of Care and Use of Laboratory Animals of the National Institute of Nuclear Research, Approval No. 02-2018).

Biodistribution and pharmacokinetic studies of ^177^Lu-scFvD2B in healthy mice were carried out by injection of 0.1 mL (3.7 MBq and 0.57 nmol) into the tail vein of 8-week-old nude male mice. Groups of animals (n = 3) were then sacrificed at 0.5, 3, 24 and 192 h post-injection. Selected organs (heart, lung, liver, pancreas, spleen and kidneys) and tissue samples (intestine, muscle, bone and blood) were dissected, weighed and measured with a NaI(Tl) crystal scintillation detector. The activity of the injected dose of ^177^Lu-scFvD2B solution was simultaneously measured in order to calculate the uptake as percentage of the injected dose per gram of tissue (%ID/g). Biodistribution data expressed as percentage of injected dose per organ (%ID/organ) was used as input for the CoKiMo spreadsheet software^22^ to obtain the organ time-activity curves. ^177^Lu-scFvD2B biological mean residence time (MRT) was computed by integration of the curve obtained for each organ. Finally, ^177^Lu-scFvD2B effective MRT was calculated by integration of the main organ activity curves, corrected by the physical decay of Lu-177.

A pulmonary microtumors mice model was used to assess the potential of ^177^Lu-scFvD2B uptake in tumors at micro-size level. Imaging studies were carried out inducing pulmonary microtumors in two groups of 8-week-old nude male mice (n = 3), by injection of 300,000 LNCaP or PC-3 cells (in 0.2 mL of phosphate-buffered saline) into the tail vein. A week later, tumor-bearing mice were injected with ^177^Lu-scFvD2B (0.1 mL; 3.7 MBq), and under 2% isoflurane anesthesia, were placed in the animal-scanner (Albira, ONCOVISION, Spain). First, a computed tomography (CT) scan was achieved with 35 kV sure voltage and 700 µA to acquire an anatomical reference. Subsequently, single-photon-emission computed tomography (SPECT) scans were obtained at 0.5, 3, 6, 24, 48, 72 and 192 h after intravenous (i.v.) administration, using a field of view of 60 mm, set at 208 keV (symmetric 20% window). Then, images were obtained by dataset reconstruction using the Ordered Subset Expectation Maximization, OSEM, algorithm. The region of interest of the tumor, main organs and whole body were drawn for each time point. PMOD 3.8 (Technologies Ltd, Switzerland) software was used to obtain quantitative data from images, and the OsiriX MD (Pixmeo SARL, Switzerland) software was employed for 3D image reconstruction.These activity data, the weight of each mouse and the time of administration and acquisition (for decay correction), were used by the PMOD software to calculate the standardized uptake values (SUV) (see Supplementary Information Figs. S1–S7). Tumor SUVs were used to generate the tumor kinetic curves and to calculate the tumor/heart and tumor/muscle ratios. Finally, mice were sacrificed after the last scan (192 h); blood and selected organs were collected and measured in order to calculate the % ID/g of tissue. For comparative purposes and as a positive control, studies on mice bearing LNCaP pulmonary tumors treated with ^177^Lu-iPSMA were also carried out as described above.

### Statistical analysis

The results are expressed as the mean ± standard deviation of the indicated determinations numbers. *In vitro* cancer cell uptake and internalization of ^177^Lu-scFvD2B, ^177^Lu-iPSMA and ^177^Lu-PSMA-617 were compared with GraphPad Prism software using two-way ANOVA test, p ≤ 0.05 was considered to be a significant difference.

## Results

### Synthesis of the DOTA-scFvD2B conjugate

**Figure 1:**
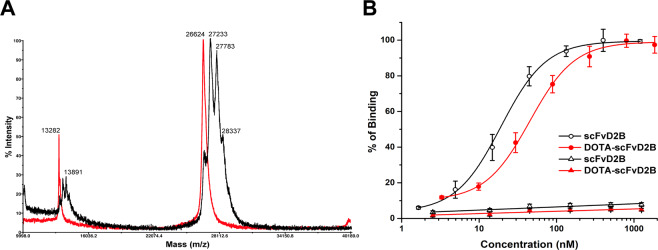
(**A**) Overlapping of MALDI-MS spectra of the unconjugated scFvD2B (red line) and DOTA-scFvD2B conjugate obtained, using a 1:5 (scFvD2B:DOTA) molar ratio (black line). (**B**) Binding affinity studies conducted by flow cytometry on the PSMA-positive PC-3-PIP cells (dots) and PSMA-negative PC-3 cells (triangles).

The scFvD2B was conjugated to DOTA in 0.2 M carbonate buffer pH 9.5 with different scFv:DOTA molar ratios at 37 °C. MALDI-MS analyses of all DOTA-scFvD2B conjugates showed multi-peaked spectra in all cases. These data indicated that a heterogeneous number of DOTA was conjugated to each scFvD2B molecule. The highest number of DOTA conjugated to scFvD2B was obtained using a 1:5 (scFvD2B:DOTA) molar ratio. Under this condition, a minimal amount of unconjugated scFvD2B (peak at 26,644) was found, and an average of 1.7 DOTA molecules per scFvD2B was estimated, considering the entire area of the peaks (Fig. 1A).

Binding assays on PSMA-transfected PC-3-PIP cells demonstrated that DOTA conjugation slightly reduced scFvD2B affinity for PSMA (Fig. 1B). The K_d_ for scFvD2B and DOTA-scFvD2B conjugate, obtained using a 1:5 molar ratio, were 19.1 ± 1.0 nM and 43.9 ± 1.7 nM (n = 3), respectively. The PC-3 cell line was used as a PSMA-negative control. Binding specificity for scFvD2B and DOTA-scFvD2B was confirmed by their negligible attachment to PSMA-negative PC-3 cells (Fig. 1B).

The binding specificity was also confirmed by blocking experiments on LNCaP cells analysed by flow cytometry. Indeed, when a 500 molar excess of scFvD2B or its DOTA derivative were used, a shift to the left of the fluorescent peak was observed, which was due to a clearly reduction of mAb D2B-FITC binding to LNCaP cells (see Supplementary Fig. 8S ).

### Radiolabeling and *in vitro* stability

**Figure 2:**
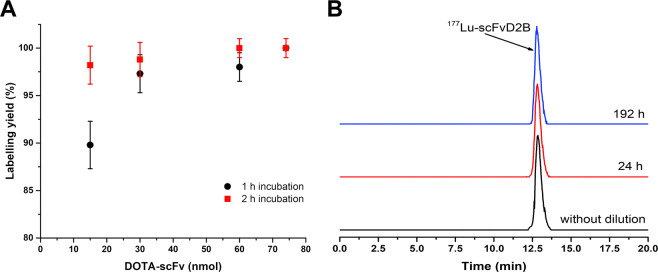
(**A**) Radiochemical yield for increasing amounts of DOTA-scFvD2B, incubated at 37 °C for 1 and 2 h. (**B**) Size-exclusion HPLC radiochromatograms of ^177^Lu-scFvD2B obtained with a molar activity of 18 MBq/nmol without dilution (black line) and after 24 and 192 h dilution in 0.1 M phosphate buffer at pH 7.0 (red and blue lines respectively).

HPLC analyses demonstrated that, to obtain ^177^Lu-scFvD2B with a radiochemical yield over 97% and molar activity up to 18 MBq/nmol, it is necessary to add at least 30 nmol of DOTA-scFvD2B to 15 µL of ^177^LuCl_3_ and incubate for 1 h at 37 °C and pH 7. However, the amount of DOTA-conjugate can be reduced if the incubation time increases to 2 h (Fig. 2A). HPLC analyses of ^177^Lu-scFvD2B after dilution in human serum or phosphate buffer confirmed its high stability. Radiochemical purity after dilution in both solutions was higher than 98% at 192 h (Fig. 2B). Radiochromatograms showed no presence of new peaks that indicate aggregation or breakdown of ^177^Lu-scFvD2B during the study period.

Radiochemical yield of both positive controls, ^177^Lu- iPSMA and ^177^Lu-PSMA-617, was higher than 98%.

### Cell binding and internalization

**Figure 3:**
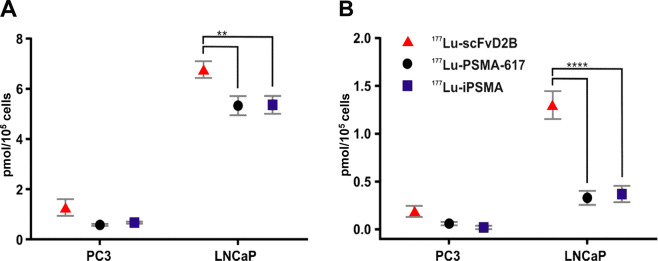
(**A**) Cell uptake and (**B**) internalization of ^177^Lu-labeled scFvD2B, ^177^Lu-PSMA-617 and ^177^Lu-iPSMA by PSMA-negative PC-3 and PSMA-positive LNCaP cells, after 2 h of incubation with 2 MBq of ^177^Lu-radiotracers (16 MBq/nmol) at 37 °C. Bars represent the average value ± SD of one experiment performed in triplicate, **p < 0.005 and ****p < 0.0001, two-way ANOVA.

Transduced PC3-PIP cells present much higher PSMA expression levels than LNCaP cells^23^. In spite of this, the *in vitro* and *in vivo* studies with ^177^Lu-scFvD2B were carried out using LNCaP cells, as in such cell line PSMA is expressed endogeneously and physiologically thus recapitulating better the real clinical setting and at the same time allowing to compare our results with the data reported in literature. *In vitro* studies showed a statistically significant higher cell uptake and internalization of ^177^Lu-scFvD2B than ^177^Lu-PSMA-617 and ^177^Lu-iPSMA in LNCaP cells (Fig. 3). The most remarkable difference between the antibody fragment and Glu-ureido based PSMA inhibitors was in cell internalization, which was at least 4 times higher for ^177^Lu-scFvD2B. This data confirmed the high *in vitro* affinity of ^177^Lu-scFvD2B for PSMA. Non-specific uptake of radiolabeled conjugate by PC-3 cells also seems to be greater than ^177^Lu-PSMA-617 and ^177^Lu-iPSMA, even if a non-statistically significant difference was found.

### Biodistribution studies of ^177^Lu-scFvD2B

**Figure 4:**
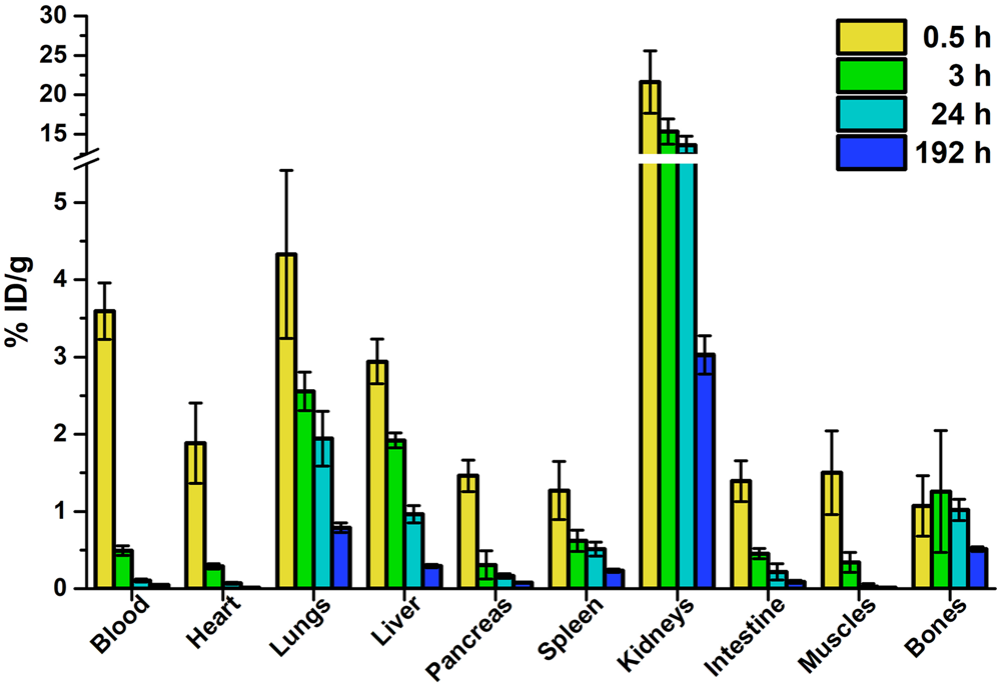
Biodistribution data (decay-corrected) up to 192 h after injection of 3.7 MBq (6.5 MBq/nmol) of ^177^Lu-scFvD2B in healthy nude male mice, and expressed as %ID/g. Bars represent the average value ± SD obtained from each group of mice (n = 3).

**Figure 5:**
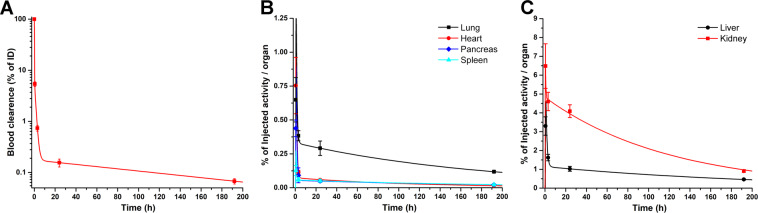
(**A**) ^177^Lu-scFvD2B blood clearance. (**B**) Biokinetic curves of non-target organs with rapid clearance. (**C**) Biokinetic curves of non-target organs with slower clearance.

*Ex-vivo* biodistribution studies were performed on healthy mice to determine the exact tissue uptake and MRT of ^177^Lu-scFvD2B in each organ. Figure 4 displays the ^177^Lu-scFvD2B biodistribution data at different time points post-injection, expressed as %ID/g. These results demonstrated that ^177^Lu-scFvD2B presents a favorable biokinetic profile, characterized by a rapid clearance from non-target tissues, as well as minimal liver accumulation. Biokinetic curves obtained from the biodistribution data, expressed as %ID/organ, demonstrated the rapid blood clearance, but also showed that kidney is the organ with the highest uptake and slowest wash-out (Fig. 5). This outcome could be explained by the predominant elimination of the scFv fragments through the renal-urinary pathway with a possible reabsorption in the proximal tubules^24^.

Values of ^177^Lu-scFvD2B biological MRT in most organs was lower than 1 hour, except for the liver and kidney, with 2.02 and 5.76 h, respectively. However, when the physical decay of lutetium-177 was considered, the effective MRT was significantly reduced in all cases (Table 1).

**Table 1:** Calculated ^177^Lu-scFvD2B biological and effective Mean Residence Times (MRT) in the main organs.

Organ	^177^Lu-scFvD2B MRT	
	Biologic (h)	Effective (h)
Blood	0.536	0.372
Heart	0.032	0.003
Lungs	0.541	0.265
Liver	2.023	1.417
Pancreas	0.069	0.011
Spleen	0.067	0.008
Kidneys	5.763	3.782

### SPECT/CT imaging studies of ^177^Lu-scFvD2B

**Figure 6:**
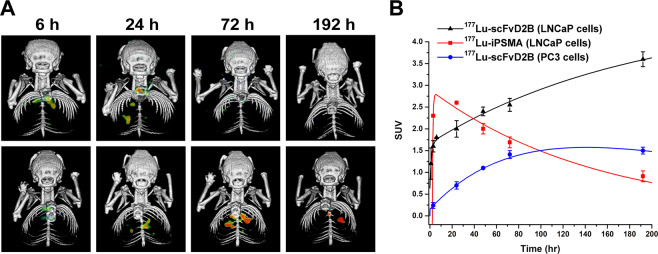
(**A**) SPECT/CT images of mice bearing LNCaP lung microtumors collected at different time points after injection of 37 MBq dose of ^177^Lu-iPSMA (upper panels) and ^177^Lu-scFvD2B (lower panels). Images are representative of one out three mice selected for each group. (**B**) ^177^Lu-scFvD2B and ^177^Lu-iPSMA tumor kinetic curves obtained from the pulmonary microtumors data. CT and SPECT single images, as well as axial and sagittal sections displaying the microtumor locations of each mouse, are shown in Supplementary Information (see Figs. S9–S32).

SPECT/CT imaging of mice bearing LNCaP pulmonary microtumors after injection of 37 MBq (6.5 MBq/nmol) dose of ^177^Lu-radiotracers clearly proved that ^177^Lu-iPSMA disclosed the fastest tumor uptake (Fig. 6A). Three hours after administration, the ^177^Lu-iPSMA tumor SUV was 2.3 and gradually increased, reaching its maximum concentration by 24 h post-injection, followed by a gradual clearance (Fig. 6B). In contrast, ^177^Lu-scFvD2B uptake in LNCaP tumors was slower, since at 3 h after injection the SUV was 1.6, but steadily rose up to a maximum value of 3.6 at 192 h (Fig. 6B). However, no statistically significant differences were found in lung weight and no appreciable morphological changes between healthy and tumor-bearing mice.

Looking at the tumor kinetic curves, it is clear that the area under the curve was larger for ^177^Lu-scFvD2B than for ^177^Lu-iPSMA, which means that more disintegrations and higher radiation absorbed doses are expected to occur in tumors treated with ^177^Lu-scFvD2B. In addition, no radioactivity in neck or head was observed that could indicate ^177^Lu-scFvD2B retention in salivary glands. The PC-3 tumor activity curve shows lower uptake of ^177^Lu-scFvD2B with faster elimination (Fig. 6B).

^177^Lu-scFvD2B organ uptake at 192 h post-injection in mice bearing LNCaP or PC-3 tumors are reported as %ID/g in Table 2. The concentration of ^177^Lu-scFvD2B in the main source organs was calculated using the SPECT imaging at 192 h, and was congruous with the %ID/g of organs obtained at the same time from the *ex-vivo* biodistribution studies in tumor-bearing mice.

**Table 2:** Uptake of ^177^Lu-scFvD2B in nude male mice bearing LNCaP or PC-3 pulmonary microtumors at 192 h post-injection.

Organ	LNCaP tumors (n = 3)	PC-3 tumors (n = 3)
Blood	0.04 ± 0.01	0.05 ± 0.00
Heart	0.03 ± 0.00	0.07 ± 0.01
Liver	0.29 ± 0.01	0.28 ± 0.03
Pancreas	0.08 ± 0.01	0.07 ± 0.01
Spleen	0.25 ± 0.04	0.22 ± 0.03
Kidneys	2.63 ± 0.22	2.99 ± 0.23
Intestine	0.12 ± 0.04	0.19 ± 0.06
Muscles	0.01 ± 0.00	0.02 ± 0.00
Bones	0.51 ± 0.06	0.39 ± 0.04
Tumor/heart*	50.55 ± 1.13	2.24 ± 0.21
Tumor/muscle*	34.51 ± 7.10	1.59 ± 0.09

Values are %ID/g, expressed as mean ± SD.

*Tumor-to-organ ratios were calculated from the SUVs obtained at 192 h.

Mice bearing micro-pulmonary tumors also showed a high kidney uptake, which slowly decreased over time, similar to what observed in healthy mice (Fig. 4). Kidney uptake of mice bearing PC-3 tumors was higher than for those bearing LNCaP tumors, although a non-statistically significant difference was found 192 h after ^177^Lu-scFvD2B injection.

The micro-pulmonary tumors (2 mm^3^) model was useful to predict the ^177^Lu-scFvD2B uptake in tumors at micro-size level. However, the tumor dimensions do not allow tissue dissection for exact determinations of the tumor uptake as %ID/g; therefore, the tumor/heart and tumor/muscle ratios were calculated using organ SUVs obtained at 192 h for LNCaP and PC-3 tumors (Table 2). Both tumor/heart and tumor/muscle ratios show to be 20 fold higher in PSMA-positive tumors with respect the negative ones.

## Discussion

Based on the encouraging results obtained with ^123^I-scFvD2B for early diagnosis of PCa^15^, this study examined the feasibility of using scFvD2B to obtain a theranostic agent for PSMA-expressing tumors. The method for the labeling of the scFvD2B fragment with ^177^Lu was described, and tumor cell uptake and internalization was studied. A healthy mice model was used to determine ^177^Lu-scFvD2B biodistribution and pharmacokinetic properties, as well as a pulmonary microtumors mouse model to compare tumor kinetics of ^177^Lu-scFvD2B and ^177^Lu-iPSMA.

^177^Lu-scFvD2B was obtained with a high radiochemical purity (>97%). Stability studies demonstrated that ^177^Lu-scFvD2B disclosed a much higher stability in solution than ^117^Lu-PSMA-617 and the albumin-binder-conjugates reported as ^177^Lu-PSMA-ALB-56 and ^177^Lu-PSMA-ALB-02^25,26^. Binding on PC-3-PIP cells performed by flow cytometry, demonstrated a scFvD2B K_d_ (19.1 ± 1.0 nM) slightly above the K_d_ reported by Frigerio *et. al*. (8.6 nM) using surface plasmon resonance (Biacore 2000)^14^. DOTA conjugation of the scFvD2B fragment, employing the 1:5 (scFvD2B:DOTA) molar ratio, reduced its affinity to 43.9 ± 1.7 nM. Nonetheless, ^177^Lu-scFvD2B showed a statistically-significant higher uptake for LNCaP cells than ^177^Lu-PSMA-617 and ^177^Lu-iPSMA, endowed with lower K_d_ values (6.33 ± 2.69 and 7.34 ± 2.97 nM, respectively)^21^. The higher ^177^Lu-scFvD2B tumor uptake could be explained by its greater internalization, which allows a higher concentration of radioactivity inside the cells.

Biodistribution studies showed that ^177^Lu-scFvD2B presents a blood clearance quite similar to that reported for ^111^In-scFvD2B^14^. Both radiometal-chelate complexes showed 0.19% ID/g in blood at 24 h after injection. This value is considerably lower than that reported for whole antibodies (from 12 to 25%ID/g)^27,28^, or for the albumin-binder-conjugated ^177^Lu-PSMA-617 as the ^177^Lu-HTK01169^23^, ^177^Lu-PSMA-ALB-56^25^ and ^177^Lu-PSMA-ALB-02^26^ (from 0.45 to 2.10%ID/g at 24 h), but higher than the ^177^Lu-PSMA-617 blood uptake (0.12 and 0.00%ID/g at 4 and 24 h, respectively)^23^. However, blood concentration of the most-promising albumin conjugates decreased quite fast 24 h after injection, and blood concentration values at 192 h were quite similar to those of ^177^Lu-scFvD2B (see Supplementary Table 1 S). Consequently, ^177^Lu-scFvD2B showed two advantages: higher internalization by PSMA-expressing cells than ^177^Lu-labeled urea derivatives, and lower radiation dose delivered to the non-target tissues, compared to ^177^Lu-anti-PSMA antibodies.

Biodistribution of ^177^Lu-scFvD2B and ^111^In-NOTA-scFvD2B derivatives showed significant differences in liver uptake values. ^111^In-scFvD2B hepatic uptake at 3 and 24 h (19.48 ± 13.91% and 22.89 ± 12.42% ID/g, respectively)^14^ was 20-fold greater than that for ^177^Lu-scFvD2B (0.95 ± 0.04% ID/g at 3 h and 0.60 ± 0.03% ID/g at 24 h). This result can be explained by the fact that authors used NOTA, which is not the best chelating agent for In-111^29^. Therefore, In-111 can be released from the complex, forming insoluble metal hydroxides that non-specifically bind to high-molecular-weight biomolecules, which are then metabolized by liver and spleen. In contrast, the DOTA chelator contributes to the great stability of ^177^Lu-scFvD2B, resulting in a low liver and spleen uptake. In fact, the calculated ^177^Lu-scFvD2B liver effective MRT (1.42 h) in mice was in the same order of magnitude as the ^177^Lu-PSMA-617 effective MRT (4.46 ± 1.72 h) in humans, as reported by Khawar *et al*.^30^.

It has been reported that murine PSMA, a 752-aminoacids glycoprotein with 91% similarity to the human PSMA 750-aminoacid sequence, is highly expressed in mouse kidneys^31^. Therefore, the high kidney uptake observed in mice upon administration of urea-based PSMA therapeutic agents might be due to the binding to the PSMA mouse isoform, likely following the interaction with the enzymatic site of PSMA proteins. Nonetheless, this would not likely represent a constrain for clinical use, since only minimal expression of PSMA has been found in human kidneys^21,23,32^. On the other hand, the high kidney accumulation of both ^177^Lu-scFvD2B and ^111^In-DTPA-D2B fragments^28^ does not likely rely on specific or non-specific binding to murine PSMA, as the administration of an excess of unlabeled antibody fragment did not modify kidney uptake. Indeed, kidney uptake is a common feature of radiolabeled scFv fragments that are endowed with a molecular weight lower than the renal clearance threshold (i.e. 60 kDa), thus leading to both renal filtration and partial re-absorption in the proximal tubules. In this regard, such limits could be partially overcome by amino acid infusions^33^ or other approaches^24^. The low kidney accumulation of ^131^I-scFvD2B reported by Frigerio *et al*.^14^ could be partly ascribable to a phenomenon of quick dehalogenation of the construct after administration^34^, leading to loss of the signal at the kidney tissue, and the release of ^131^I^16^ that in turn concentrates in the thyroid gland and stomach as showed by the SPECT/CT images^15^. Overall, in-depth biodistribution analyses in humans are mandatory to assess the real renal uptake before draining a conclusion about the potential application of scFv moieties for therapeutic purposes.

^177^Lu-scFvD2B SPECT/CT imaging in mice bearing pulmonary microtumors displayed a slow tumor uptake, which was maintained for 192 h, and a rapid kidney uptake that decreased over time. Similar outcomes were reported by Kou *et. al*. using the ^177^Lu-HTK01169^23^. Biokinetic curves demonstrated that the LNCaP tumor uptake and retention of ^177^Lu-scFvD2B was higher than for ^177^Lu-iPSMA. Similar results were found by Kou *et. al*., when compared to ^177^Lu-PSMA-617 with ^177^Lu-HTK01169^23^. These results are most likely consequences of different factors, such as the higher stability, slower blood clearance and higher tumor cell internalization of the radiotracers with higher molecular weight, as well as their metabolism in tumor cells, related to the retention of radioactivity in tumors^27^. Based on these results, it can be deduced that ^177^Lu-scFvD2B could supply a higher radiation dose to PSMA-positive tumors than ^177^Lu-iPSMA and could therefore be a better theranostic agent. Consequently, dosimetrics studies should be carried out even though the clinical translation is certainly more expensive for a labeled antibody fragment than urea derivatives. In addition, the scFvD2B ligand prepared in this research could also be conjugated to deferoxamine for the labelling with zirconium-89 for PET imaging and possible routine use of the ^89^Zr-/^177^Lu-scFvD2B as theranostic companion.

## Conclusions

The ^177^Lu-scFvD2B fragment was obtained with a high radiochemical yield and purity and showed specific recognition for PSMA-expressing tumors *in vivo* and *in vitro*. In addition, the high ^177^Lu-scFvD2B stability and internalization trigger a high and prolonged tumor uptake, providing to this agent a great potential in delivering ablative radiation doses to PSMA-expressing tumors. However, further studies must be carried out in order to evaluate the dosimetry and therapeutic efficacy of ^177^Lu-scFvD2B.

## Supplementary information

Supplementary Information.

## Data Availability

All data generated or analysed during this study are included in this published article and its Supplementary Information files.
