# Supplementary material for: Development of 177Lu-scFvD2B as a Potential Immunotheranostic Agent for Tumors Overexpressing the Prostate Specific Membrane Antigen
Source: Sci Rep. 2020 Jun 9;10:9313. doi: 10.1038/s41598-020-66285-2 (PMC7283306; doi:10.1038/s41598-020-66285-2)
Supplement: Supplementary file 1 — Supplementary Information. [file 41598_2020_66285_MOESM1_ESM.pdf]

Supplementary Information

Development of ^{177}Lu-scFvD2B as a Potential Immunotheranostic Agent for Tumors Overexpressing the Prostate Specific Membrane Antigen

Debora Carpanese[1], Guillermina Ferro-Flores[2], Blanca Ocampo-Garcia[2], Clara Santos-Cuevas[2], Nicola Salvarese[3], Mariangela Figini[4], Giulio Fracasso*[5], Laura De Nardo[6], Cristina Bolzati[3], Antonio Rosato[†1,7] and Laura Meléndez-Alafort*[,†1].

[1]Istituto Oncologico Veneto IOV-IRCCS, Padua, Italy; [2]Laboratorio Nacional de Investigación y Desarrollo de Radiofármacos-CONACyT, Instituto Nacional de Investigaciones Nucleares, Estado de México, Mexico; [3]Institute of Condensed Matter Chemistry and Energy Technologies, National Research Council, Padua, Italy; [4]Biomarker Unit, Dipartimento di Ricerca Applicata e Sviluppo Tecnologico, Fondazione IRCCS Istituto Nazionale dei Tumori, Milan, Italy; [5]Department of Medicine, University of Verona, Verona, Italy; [6]Department of Physics and Astronomy, University of Padua, Padua Italy; [7]Department of Surgery, Oncology, and Gastroenterology, University of Padua, Padua Italy

*Co-corresponding authors

[†]Co-senior authors

CORRESPONDENCE TO:

Laura Meléndez-Alafort

Istituto Oncologico Veneto IOV-IRCCS, Via Gattamelata 64, 35138 Padova, Italy

Tel (+39) 049/8830600, Fax (+39) 049/641925

e-mail: laura.melendezalafort@iov.veneto.it, lmalafort@yahoo.com

Giulio Fracasso

University of Verona, Piaz.le Scuro 10, 37134 Verona Italy

Tel (+39) 045/8126457, Fax (+39) 045/8126455,

e-mail: giulio.fracasso@univr.it

Table S1. Comparison of blood concentration vs time of the most-promising albumin-binder-conjugated ^{177}Lu-PSMA-617 reported and ^{177}Lu-scFvD2B (decay-corrected values expressed as %ID/g, mean ± SD).

Complex	4h	24h	96 h	192h	Reference
^{177}Lu-PSMA-ALB-56	16.2 ± 1.40	1.29 ± 0.28	0.36 ± 0.24	0.09 ± 0.08	[Umbricht *et al* Mol. Pharm. **2018**, *15*: 2297]
^{177}Lu-PSMA-ALB-02	3.05 ± 0.33	0.45 ± 0.09	0.14 ± 0.03	0.05 ± 0.01	[Benesova *et al* Mol. Pharm. **2018**, *15*: 934]
^{177}Lu-HTK01169	10.2 ± 1.40	2.10 ± 0.41	0.28 ± 0.10 (72h)	0.06 ± 0.03 (120 h)	[Kuo *et al*, Mol. Pharm. **2018**, *15*: 5183]
^{177}Lu-scFvD2B	0.49 ± 0.06 (3 h)	0.10 ± 0.02	--------	0.05 ± 0.01	This manuscript.

Determination of Standardized Uptake Values (SUV)

The quantitative tumor uptake was reported from the images through the SUV calculated as follows: in the axial sections of SPECT, the two-dimensional regions of interest are drawn to form a volume of interest (VOI). The program calculates the dimensions of that volume and the following data must be entered: mouse size, weight and sex, radionuclide used (for decay correction), activity in the pre-injection syringe and the date and time of measurement, date and injection time, residual activity in the syringe and date and time of said measurement, and date and time of image acquisition. With this data, the program calculates the SUV (ratio of the image-derived radioactivity concentration c_{img} and the whole-body concentration of the injected radioactivity c_{inj}). This program requests a calibration, which is carried out with a phantom, with known volume and activity of the studied radionuclide.

Figures S1 to S7 show and example of CT and SPECT image fusion with axial, coronal and sagittal views for the micrometastasis localization and the general procedure to obtain quantitative data from images by using the PMOD 3.8 software.

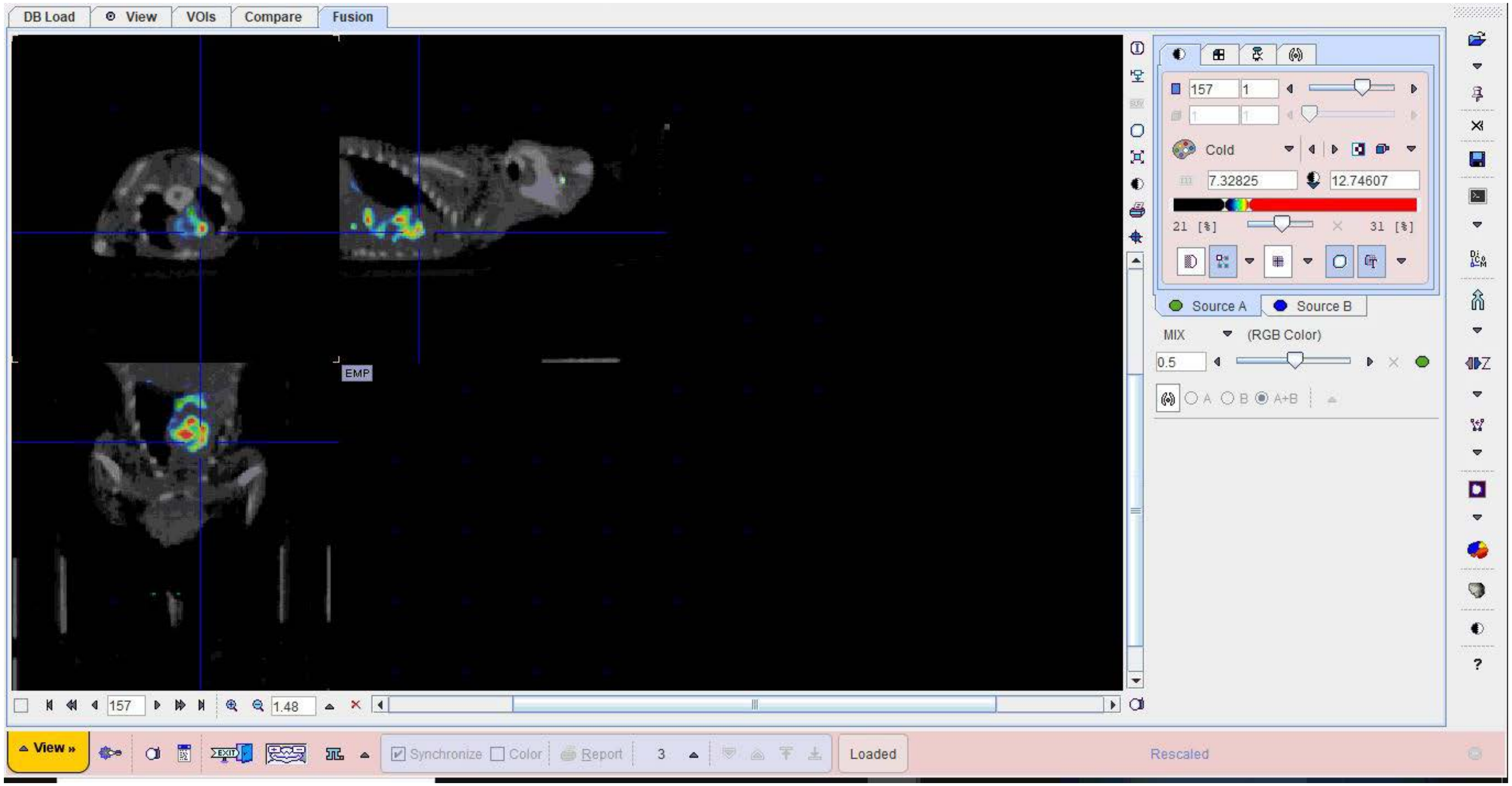

Figure S1. PMOD example of CT and SPECT image fusion with axial, coronal and sagittal views for the micrometastasis localization.

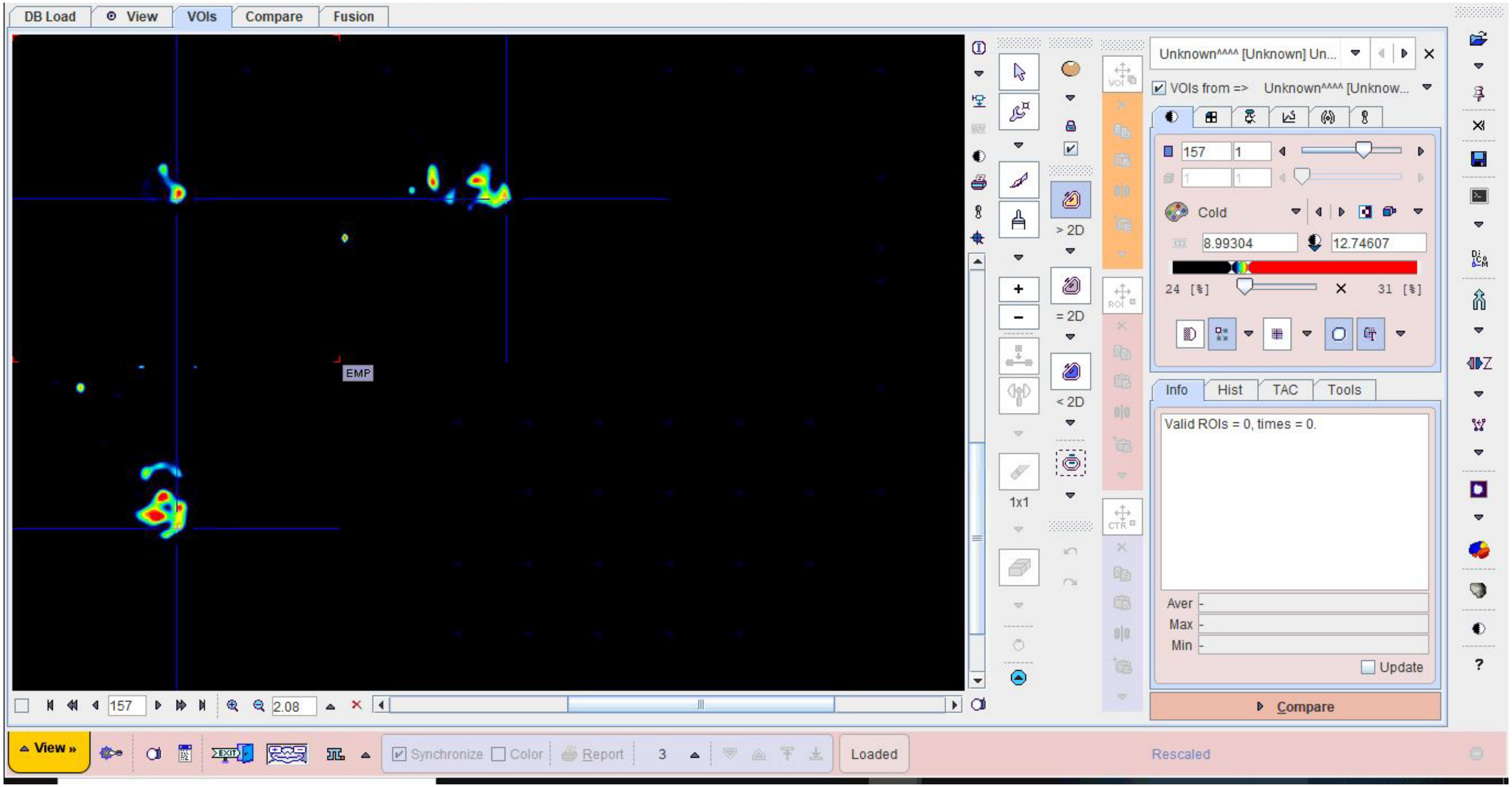

Figure S2. PMOD example of SPECT image with axial, coronal and sagittal views for the ROI selection.

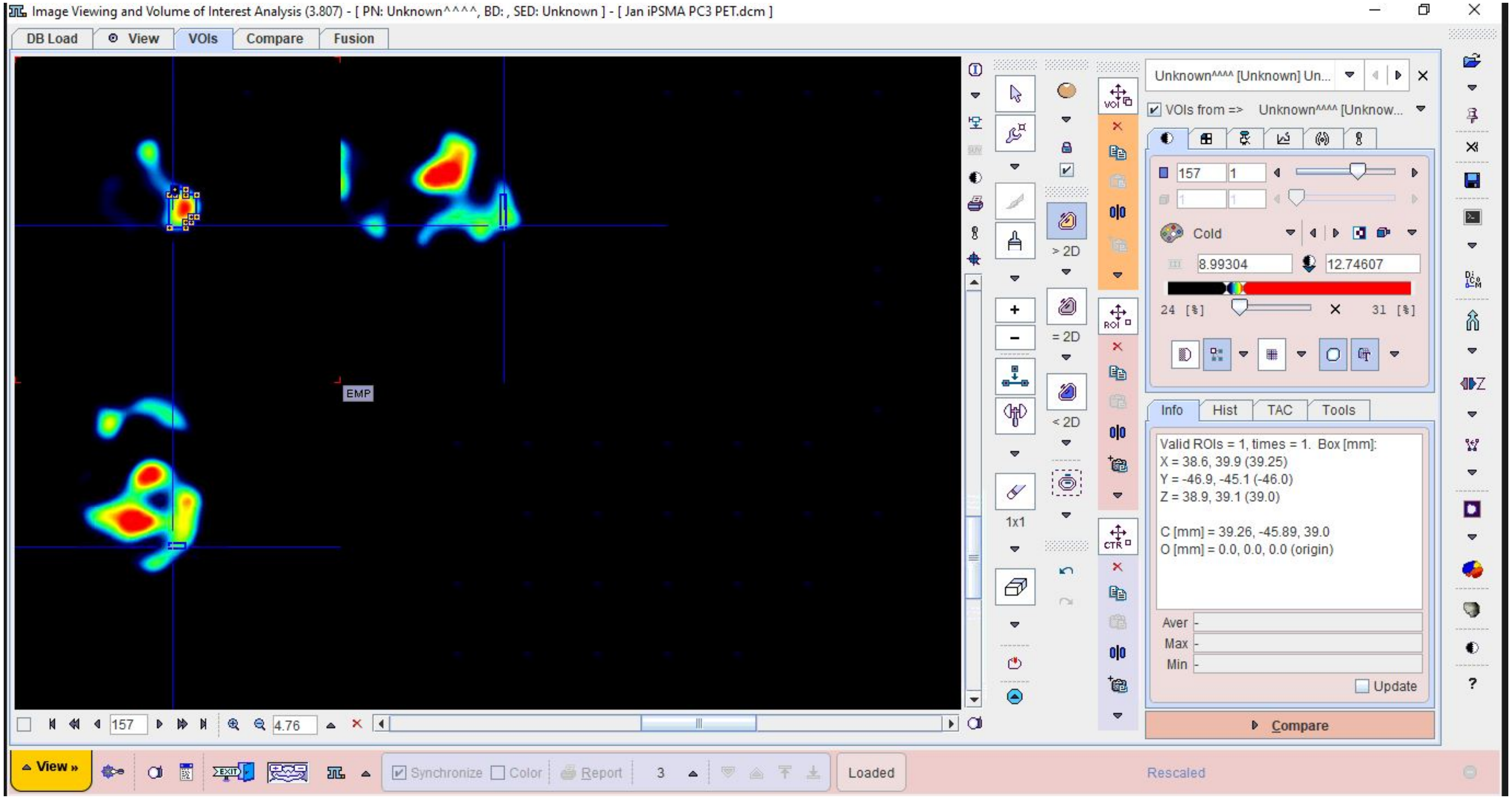

Figure S3. PMOD example of SPECT image with axial, coronal and sagittal views with a ROI selection.

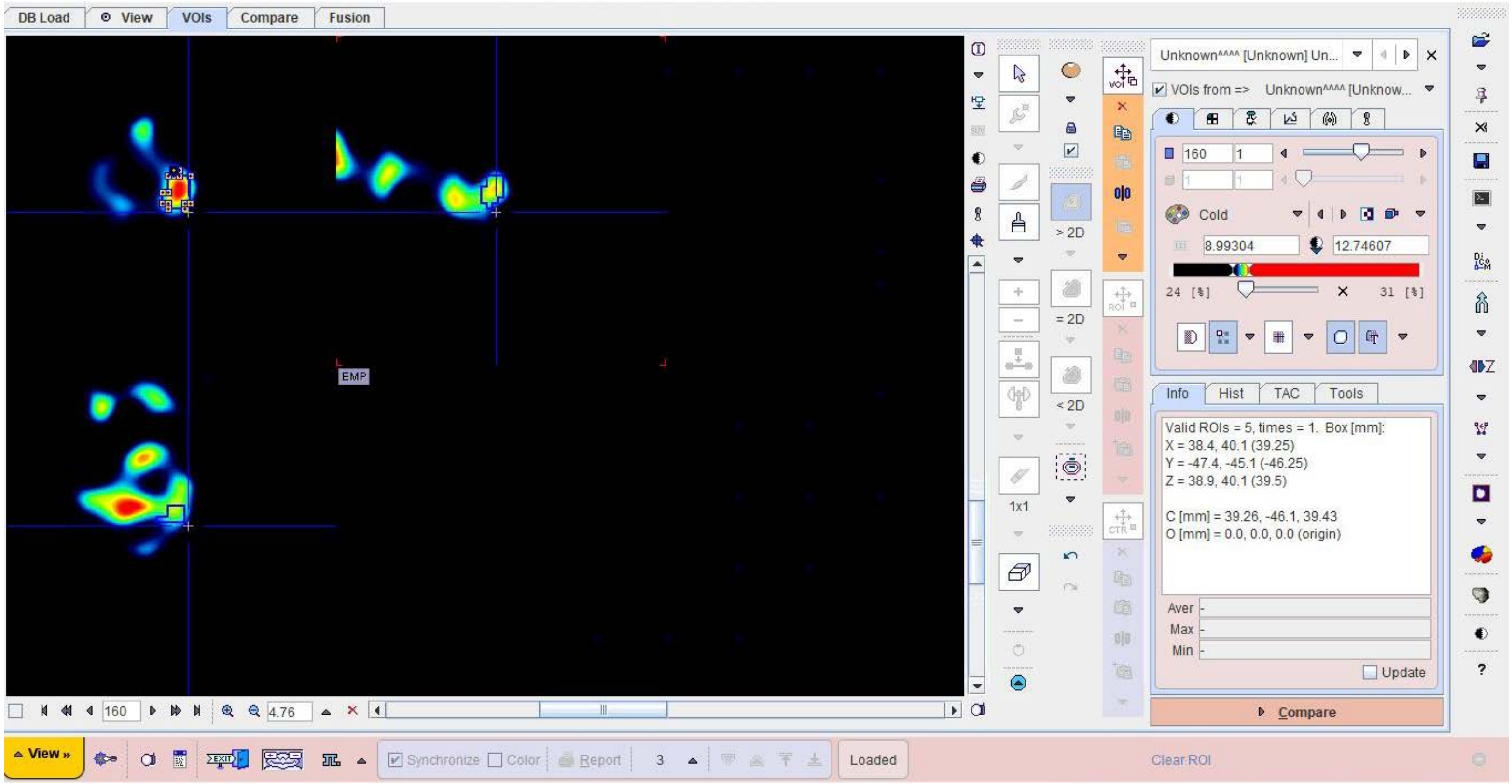

Figure S4. PMOD example of SPECT image with axial, coronal and sagittal views with a VOI selection.

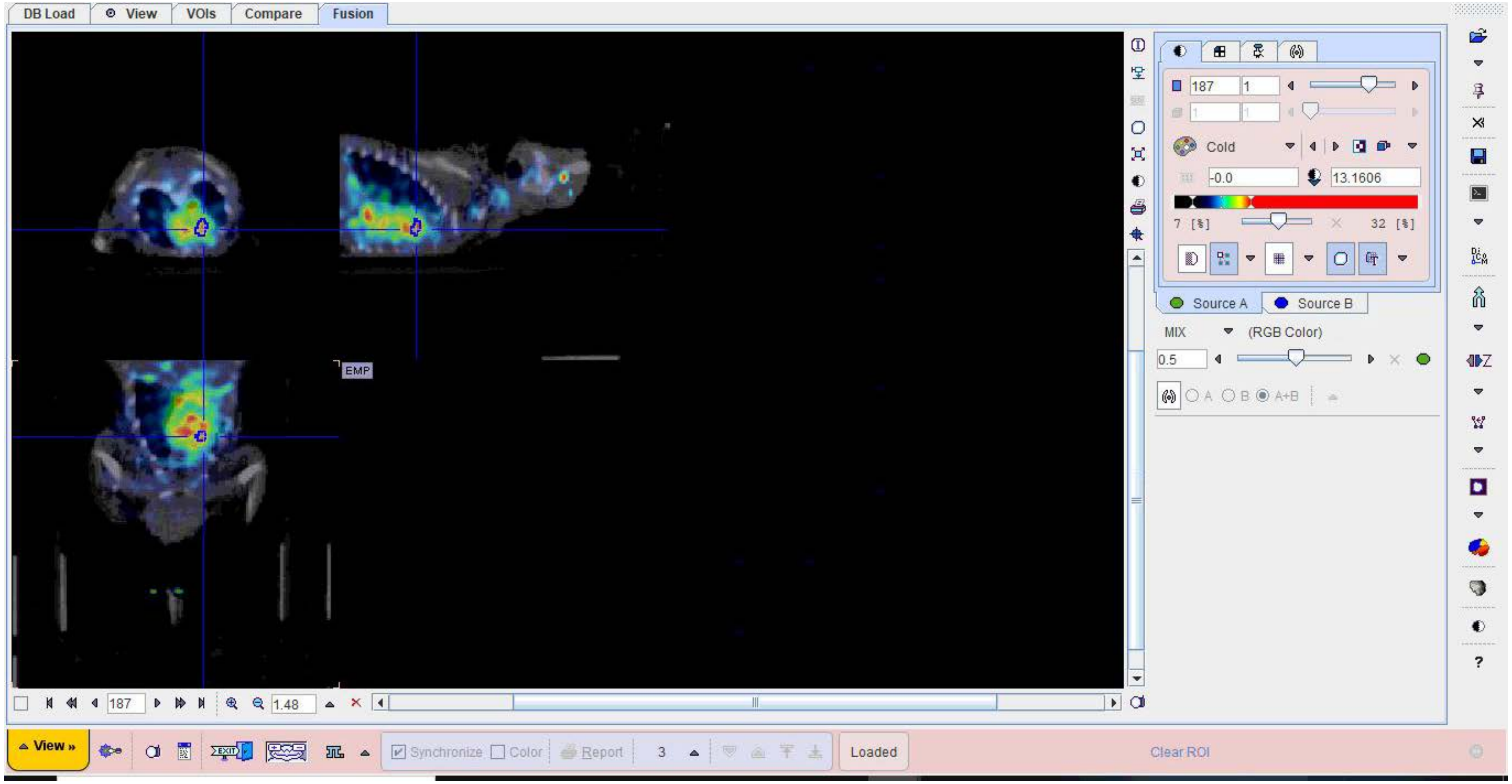

Figure S5. PMOD example of SPECT/CT image with axial, coronal and sagittal views with a VOI selection.

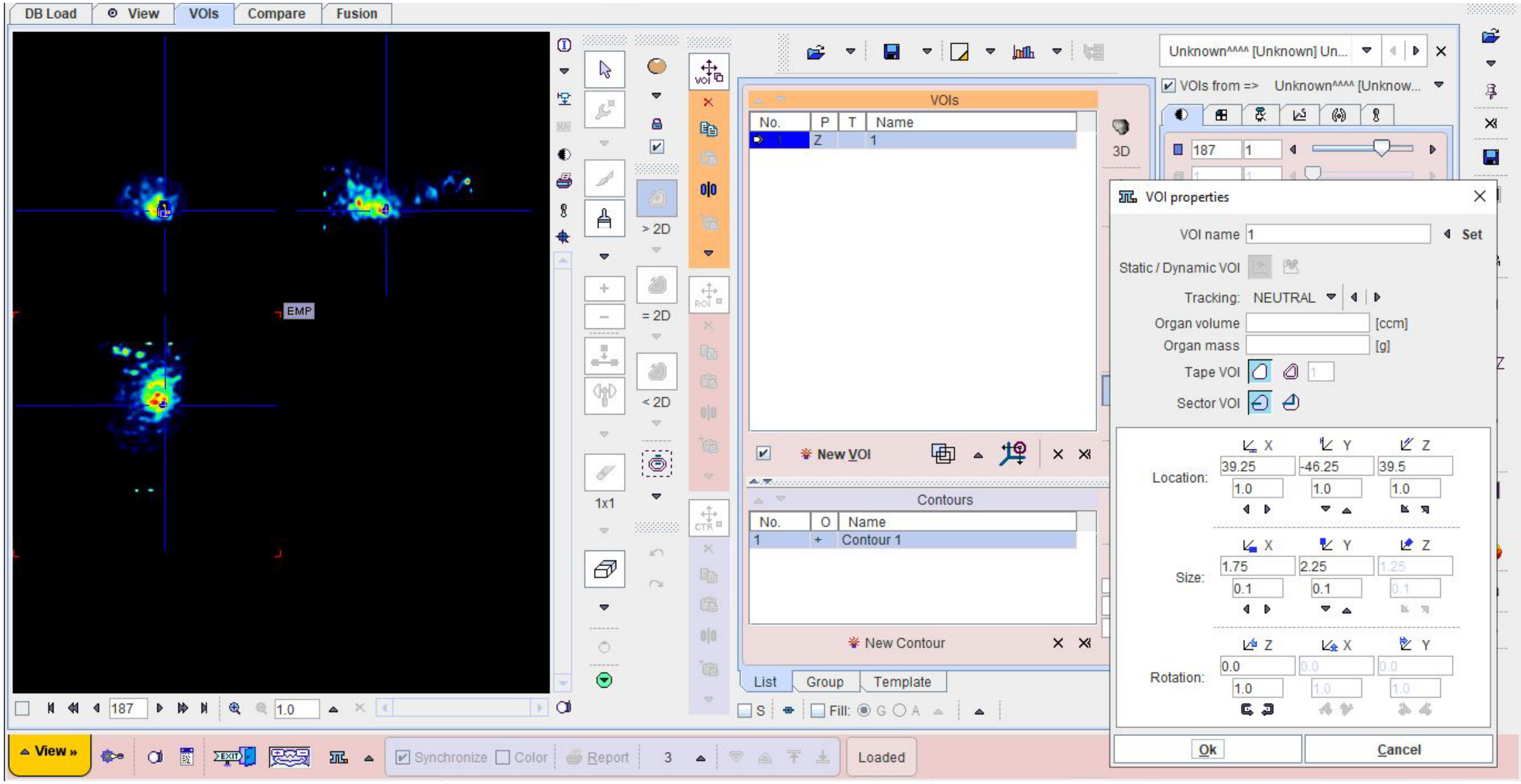

Figure S6. PMOD example of SPECT image with axial, coronal and sagittal views with a VOI selection and VOI data.

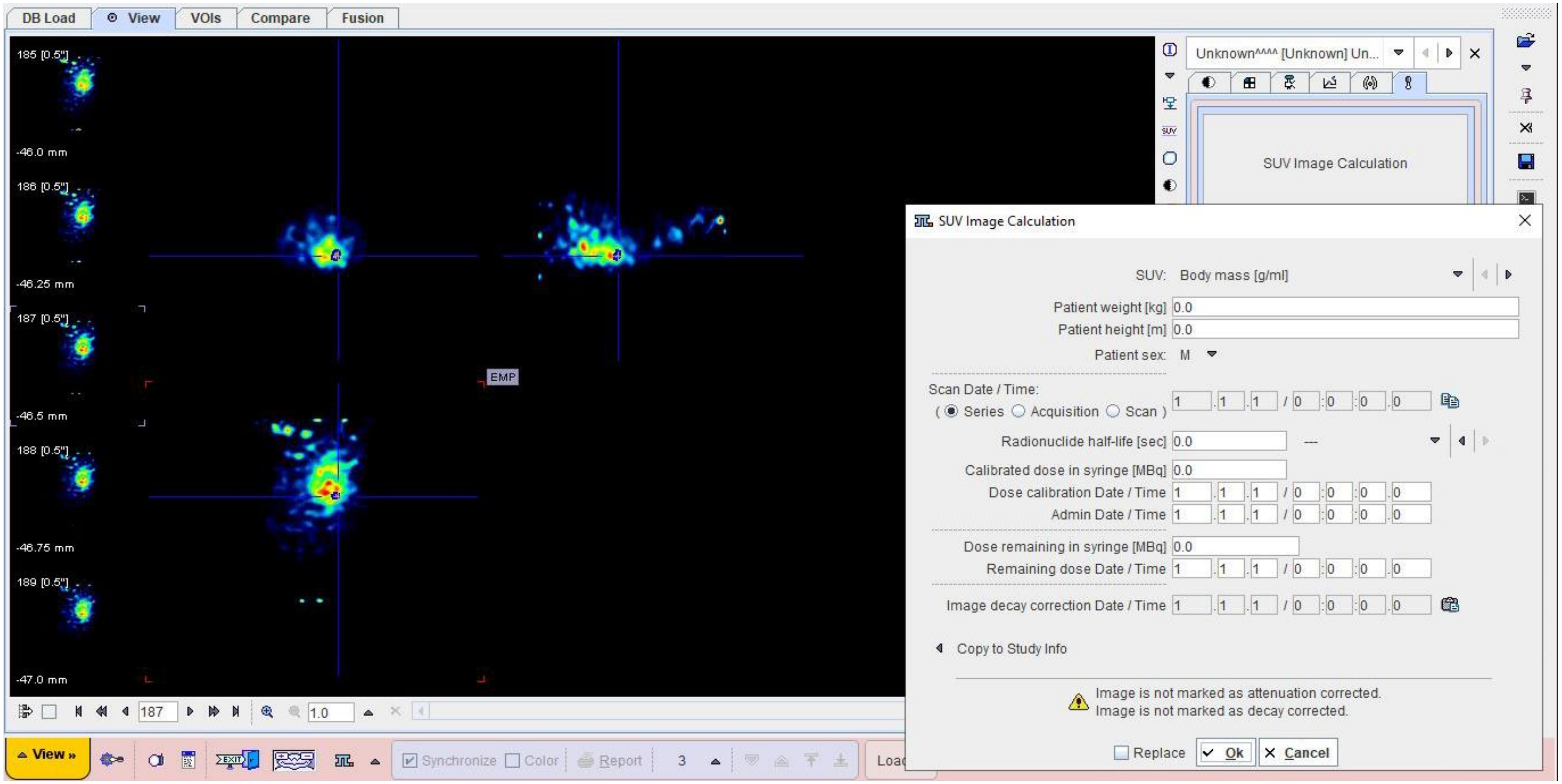

Figure S7. PMOD example of SPECT image with axial, coronal and sagittal views with a VOI selection and window example of the information required for SUV calculation.

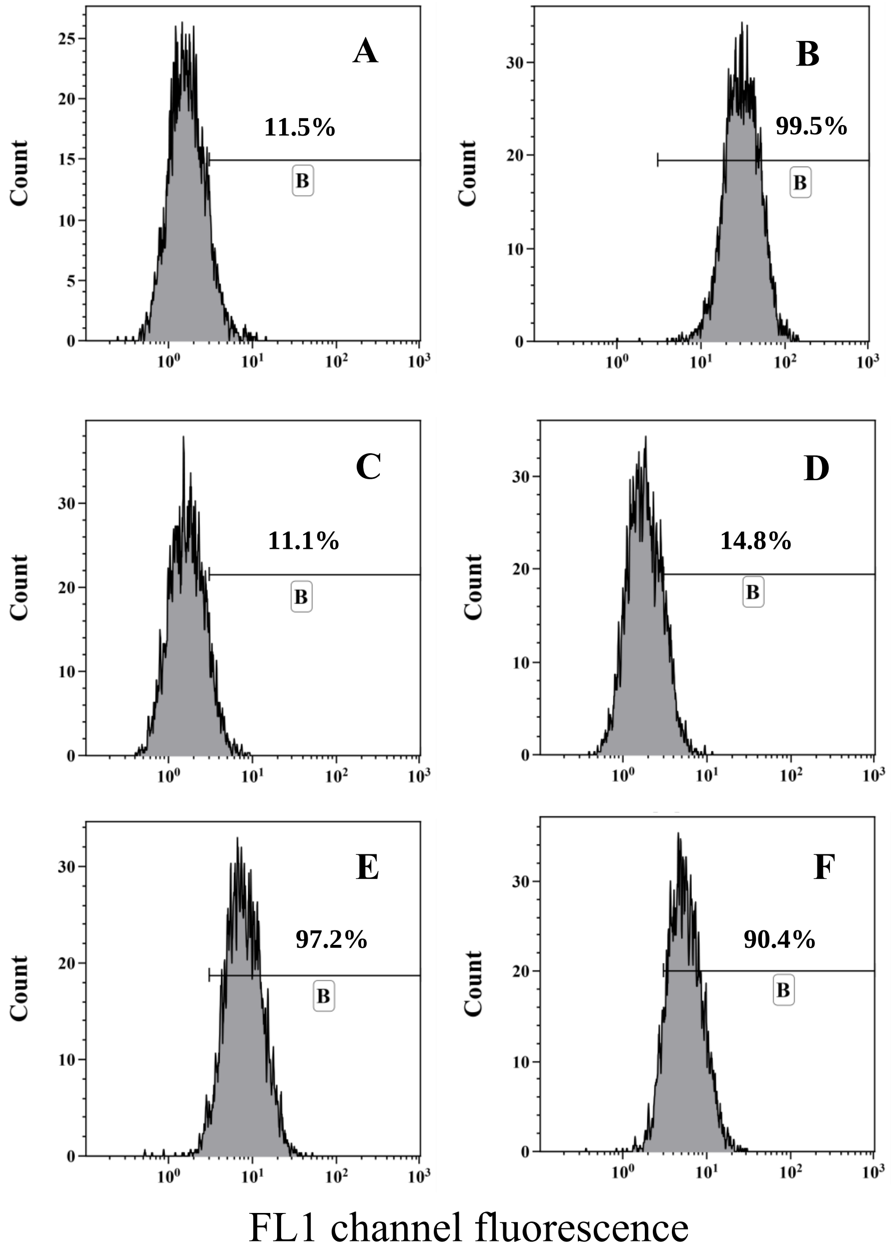

Figure 8S. Flow cytometry analysis of PSMA antigen blocking; scFvD2B or its DOTA derivative were used to hinder FITC-labeled mAb D2B binding to LNCaP cells. Cells were incubate with (A) isotype control FITC-labeled Ab, (B) 0.1 ug of FITC-labeled mAb D2B, (C and E) 500 or 5 molar excess of scFvD2B or (D and F) 500 or 5 molar excess of DOTA-scFvD2B. Data are depicted by the guassian fluorescence curves obtained from 4,000 cells. Stained cells were gated and their percentage showed in each histrogram.

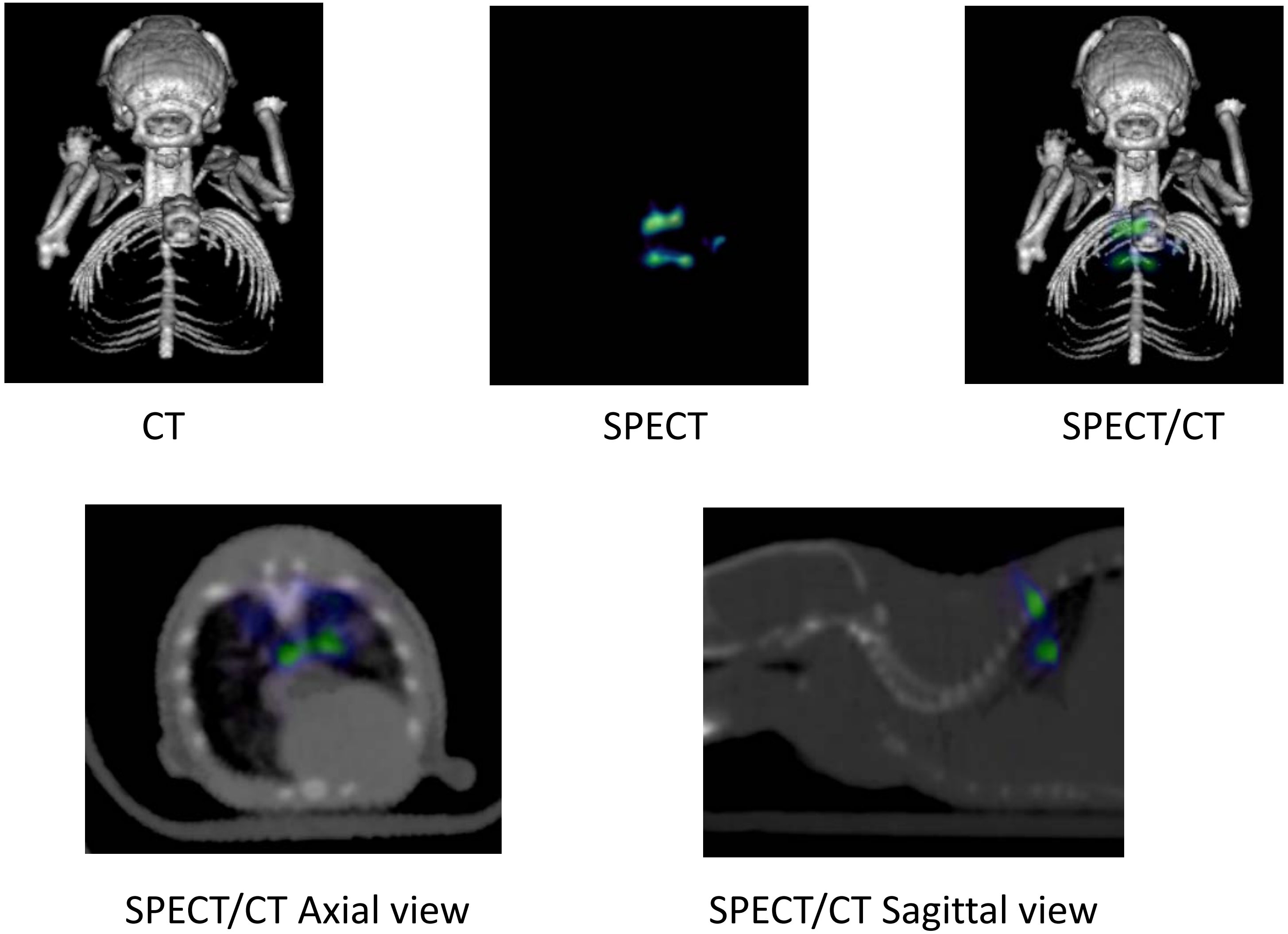

FIGURE S9. CT, SPECT, SPECT/CT, Axial and Sagittal view images of ^{177}Lu-scFvD2B in mouse No.1 bearing LNCaP micro-pulmonary tumors, collected at 6 h p.i.

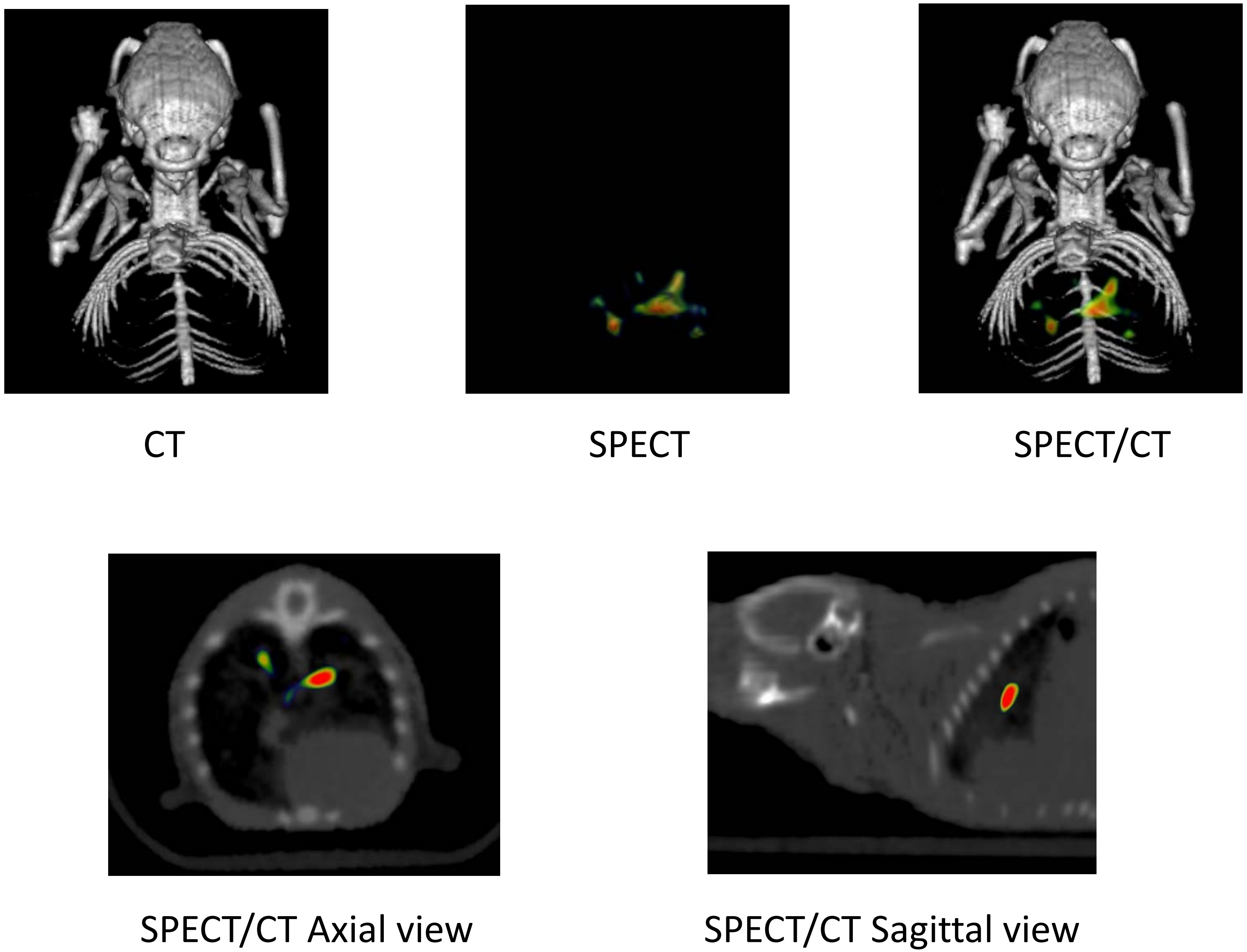

FIGURE S10. CT, SPECT, SPECT/CT, Axial and Sagittal view images of ^{177}Lu-scFvD2B in mouse No.1 bearing LNCaP micro-pulmonary tumors, collected at 24 h p.i.

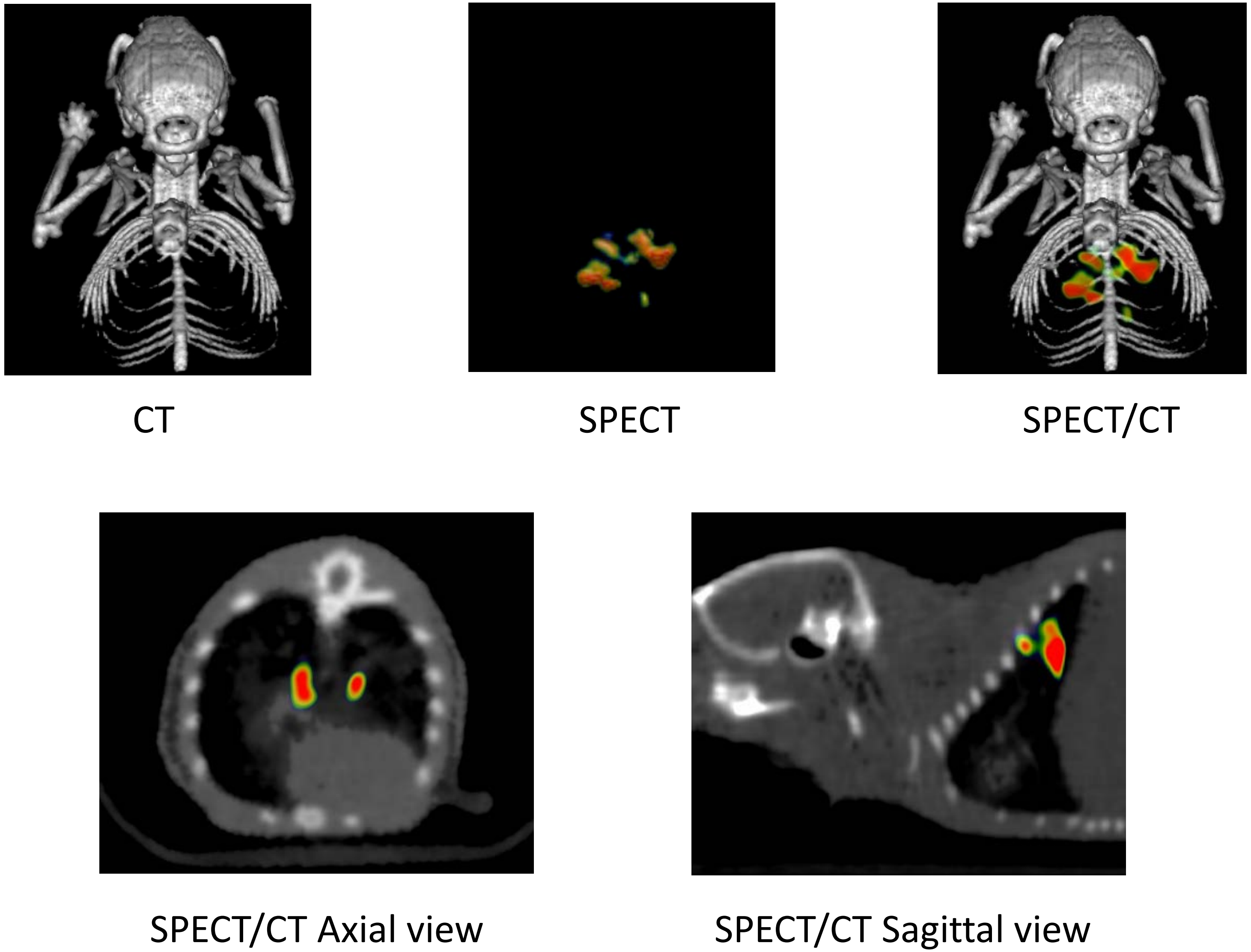

FIGURE S11. CT, SPECT, SPECT/CT, Axial and Sagittal view images of ^{177}Lu-scFvD2B in mouse No.1 bearing LNCaP micro-pulmonary tumors, collected at 72 h p.i.

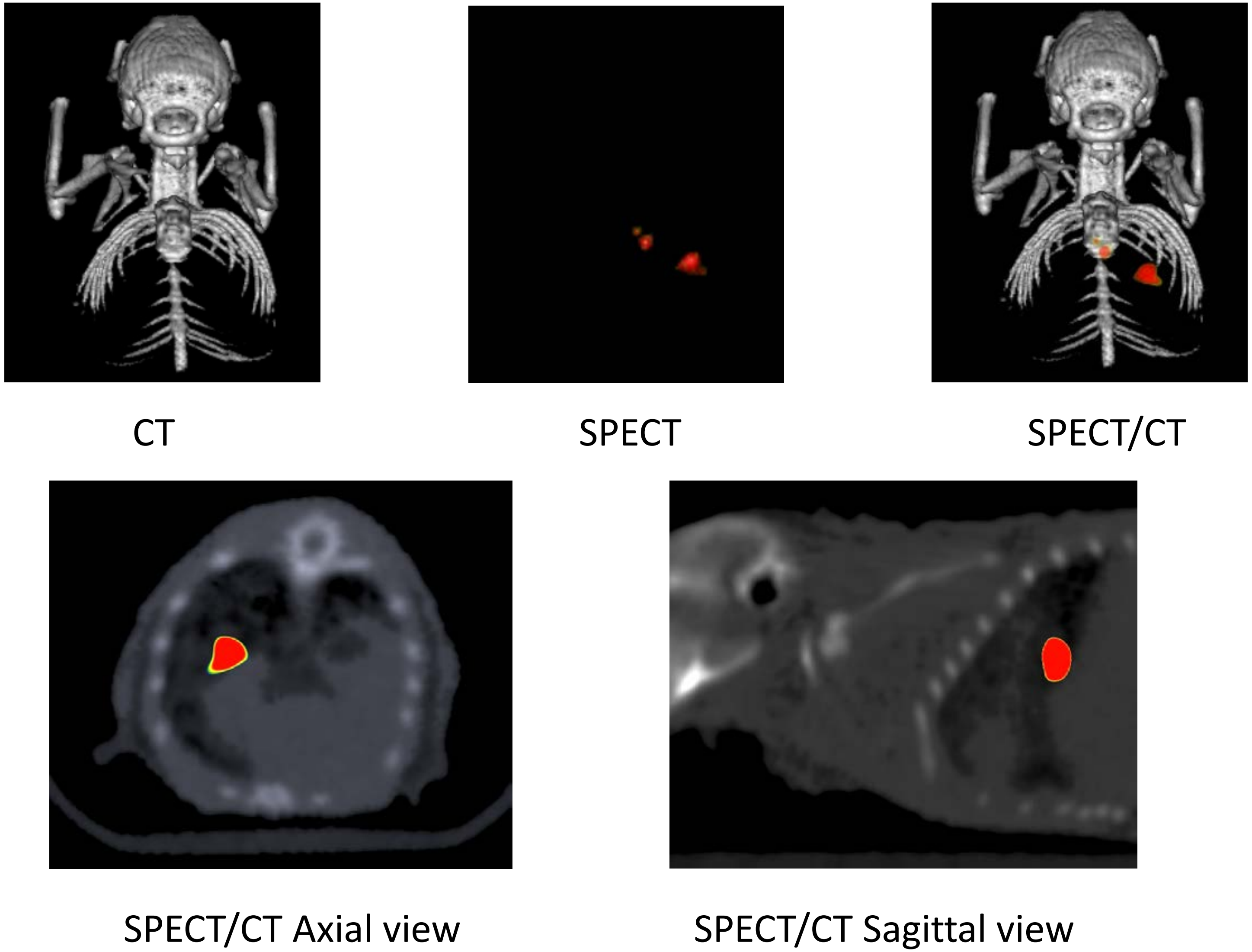

FIGURE S12. CT, SPECT, SPECT/CT, Axial and Sagittal view images of ^{177}Lu-scFvD2B in mouse No.1 bearing LNCaP micro-pulmonary tumors, collected at 192 h p.i.

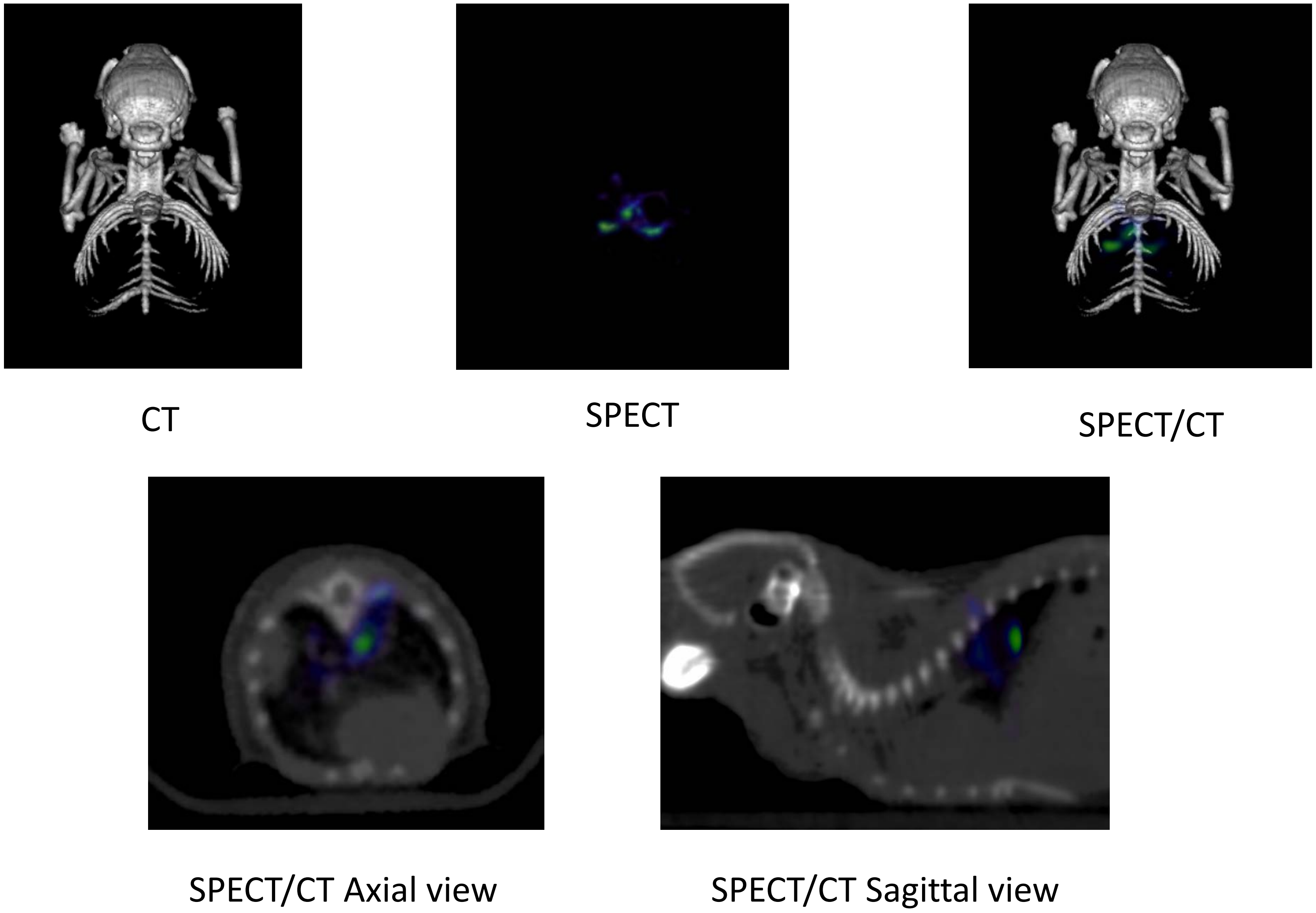

FIGURE S13. CT, SPECT, SPECT/CT, Axial and Sagittal view images of ^{177}Lu-scFvD2B in mouse No.2 bearing LNCaP micro-pulmonary tumors, collected at 6 h p.i.

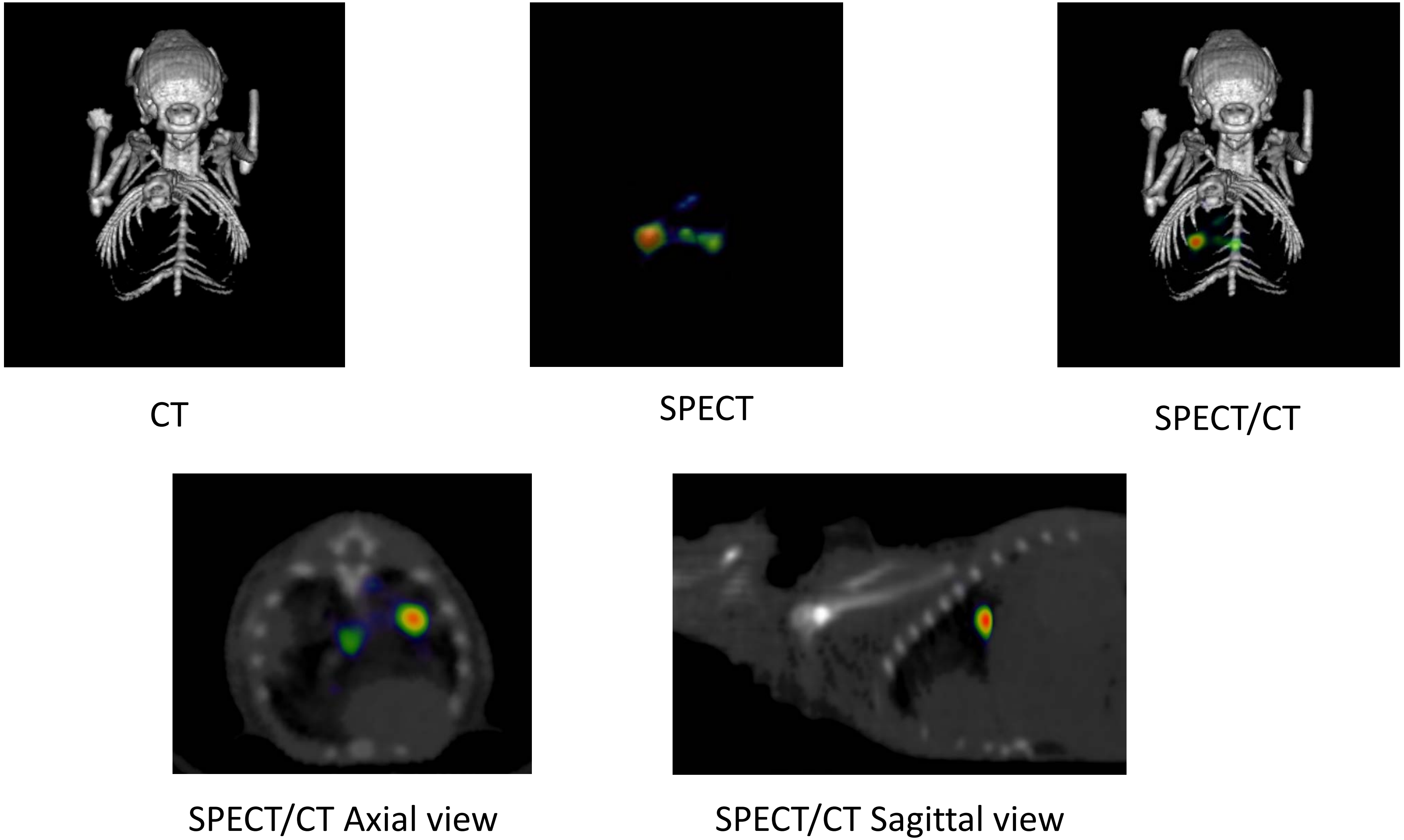

FIGURE S14. CT, SPECT, SPECT/CT, Axial and Sagittal view images of ^{177}Lu-scFvD2B in mouse No.2 bearing LNCaP micro-pulmonary tumors, collected at 24 h p.i.

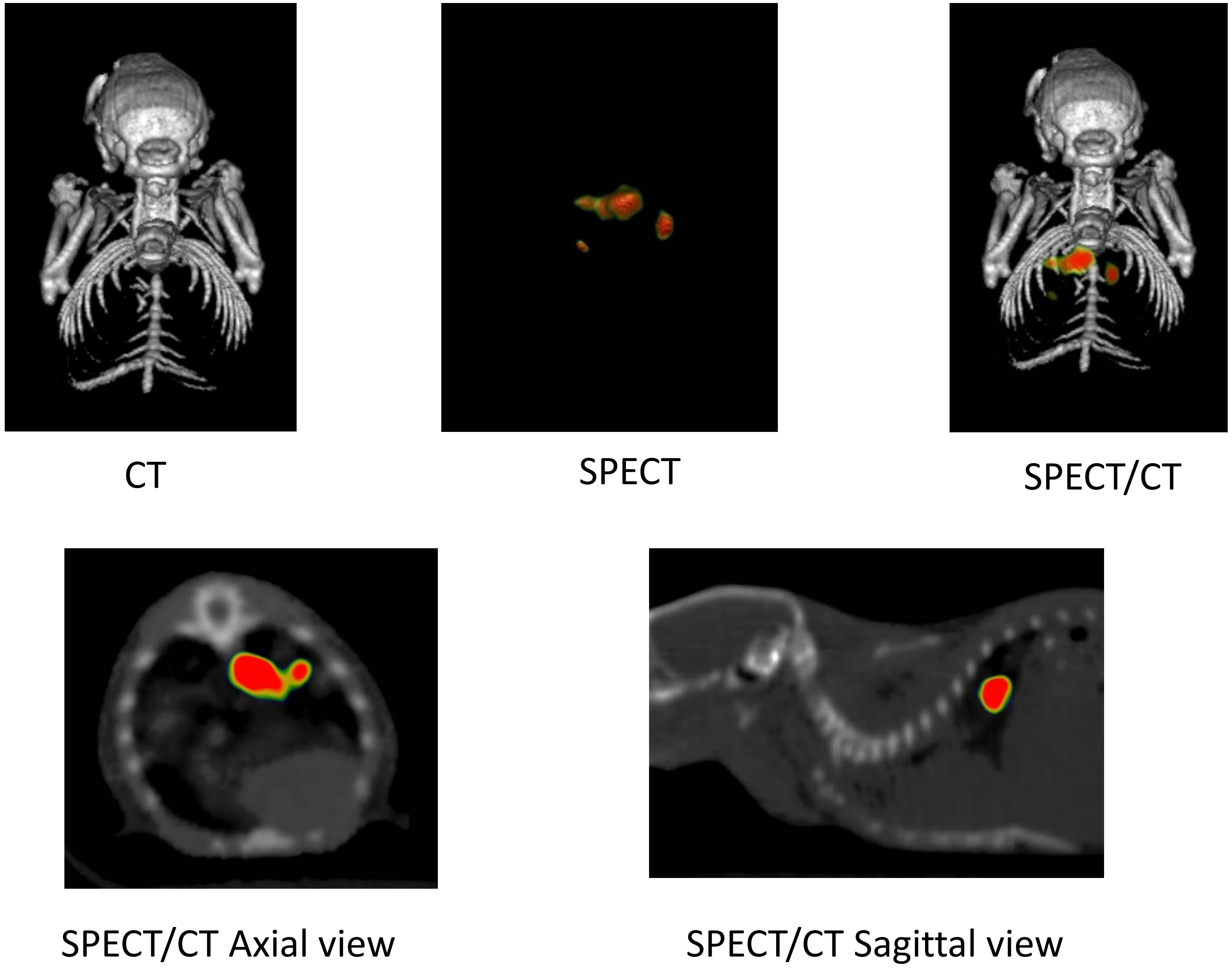

FIGURE S15. CT, SPECT, SPECT/CT, Axial and Sagittal view images of ^{177}Lu-scFvD2B in mouse No.2 bearing LNCaP micro-pulmonary tumors, collected at 72 h p.i.

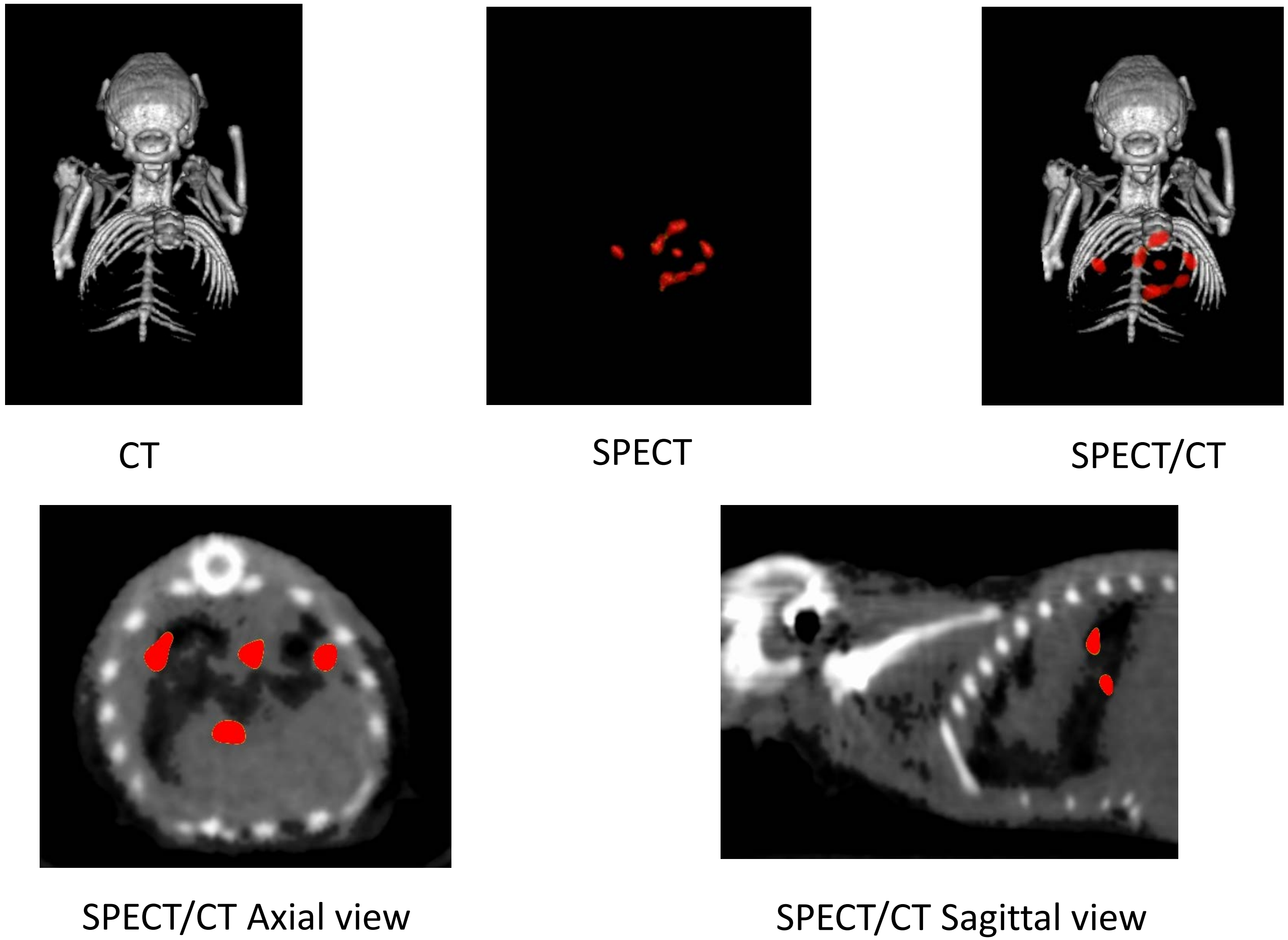

FIGURE S16. CT, SPECT, SPECT/CT, Axial and Sagittal view images of ^{177}Lu-scFvD2B in mouse No.2 bearing LNCaP micro-pulmonary tumors, collected at 192 h p.i.

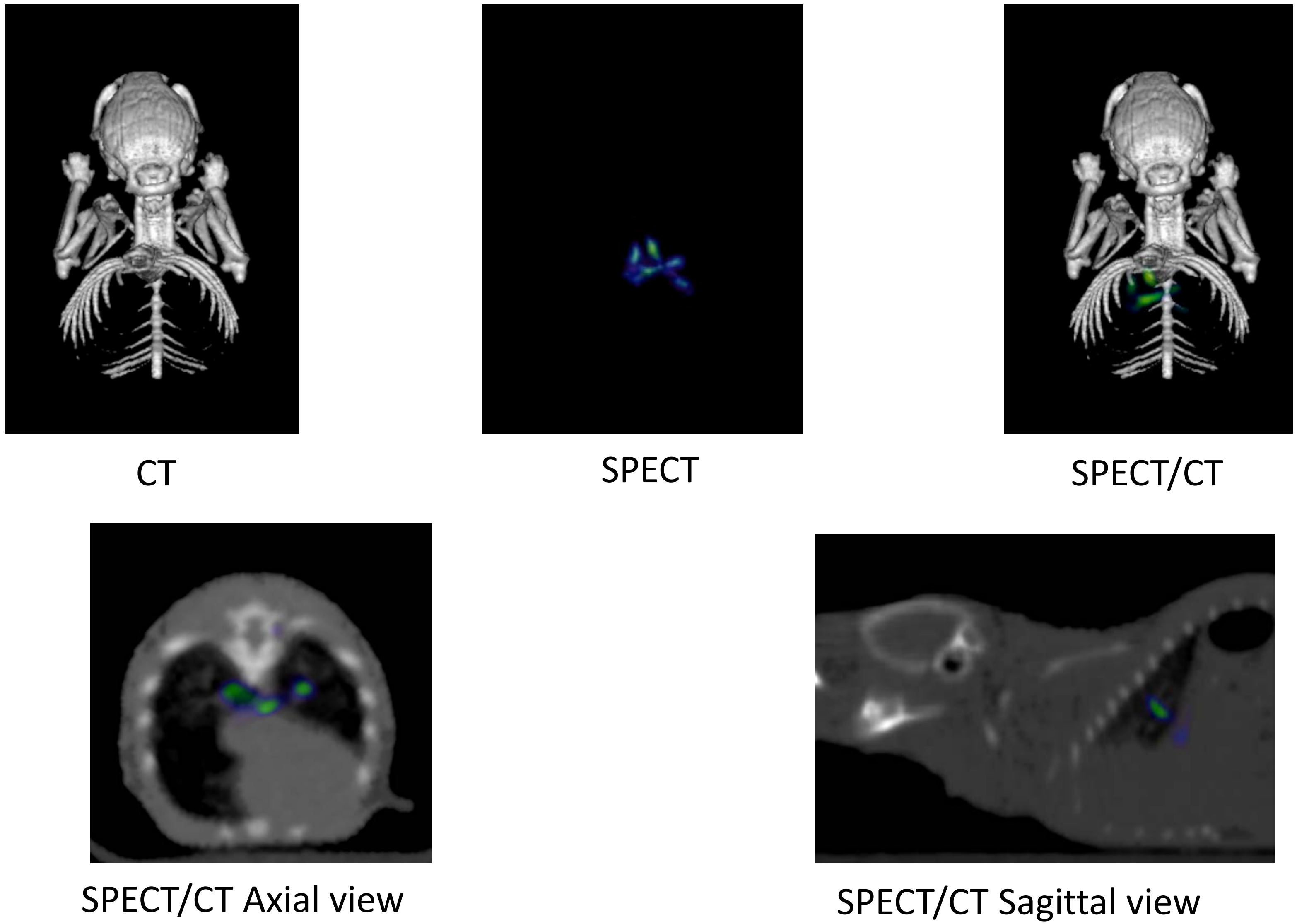

FIGURE S17. CT, SPECT, SPECT/CT, Axial and Sagittal view images of ^{177}Lu-scFvD2B in mouse No.3 bearing LNCaP micro-pulmonary tumors, collected at 6 h p.i.

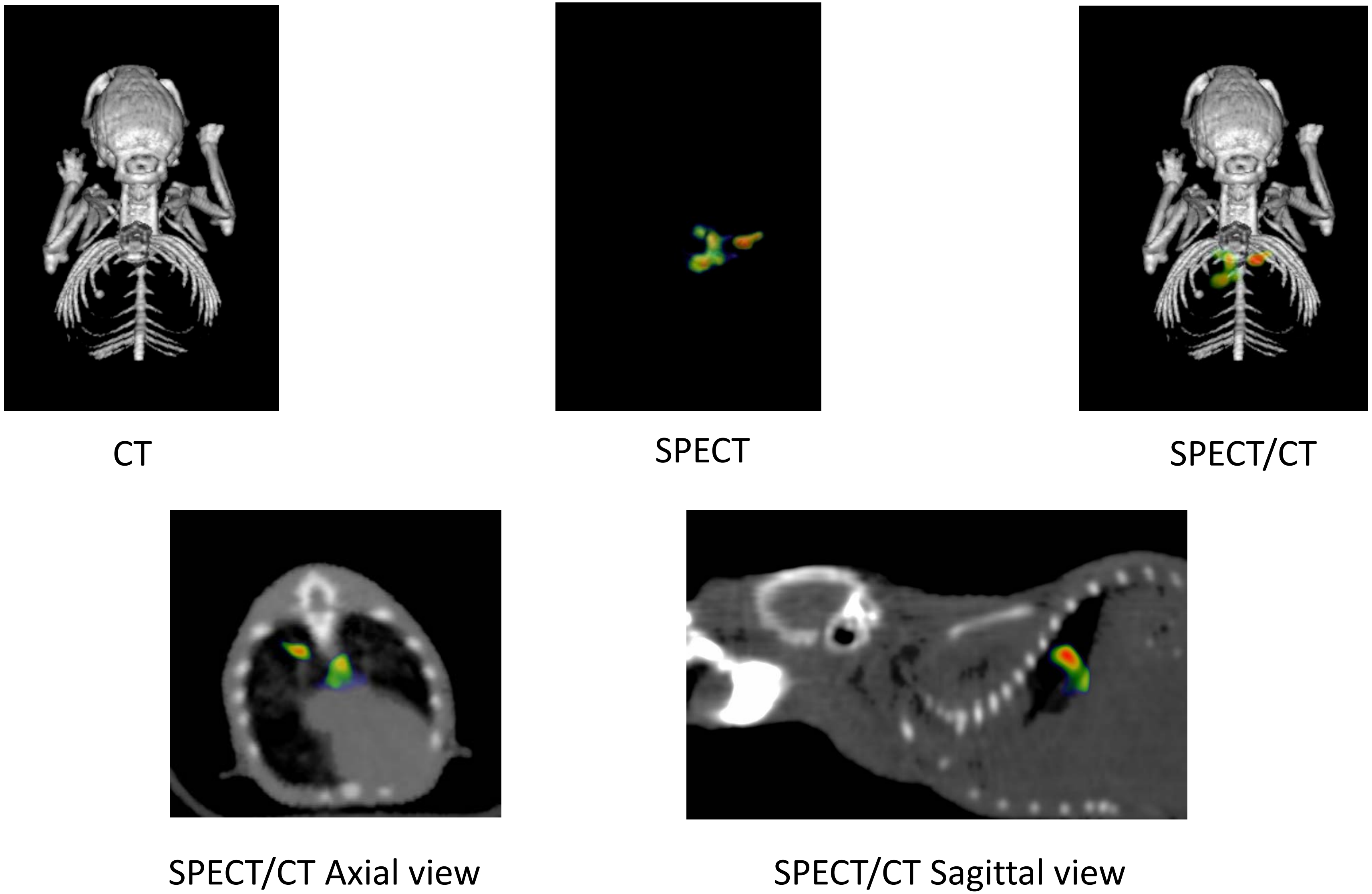

FIGURE S18. CT, SPECT, SPECT/CT, Axial and Sagittal view images of ^{177}Lu-scFvD2B in mouse No.3 bearing LNCaP micro-pulmonary tumors, collected at 24 h p.i.

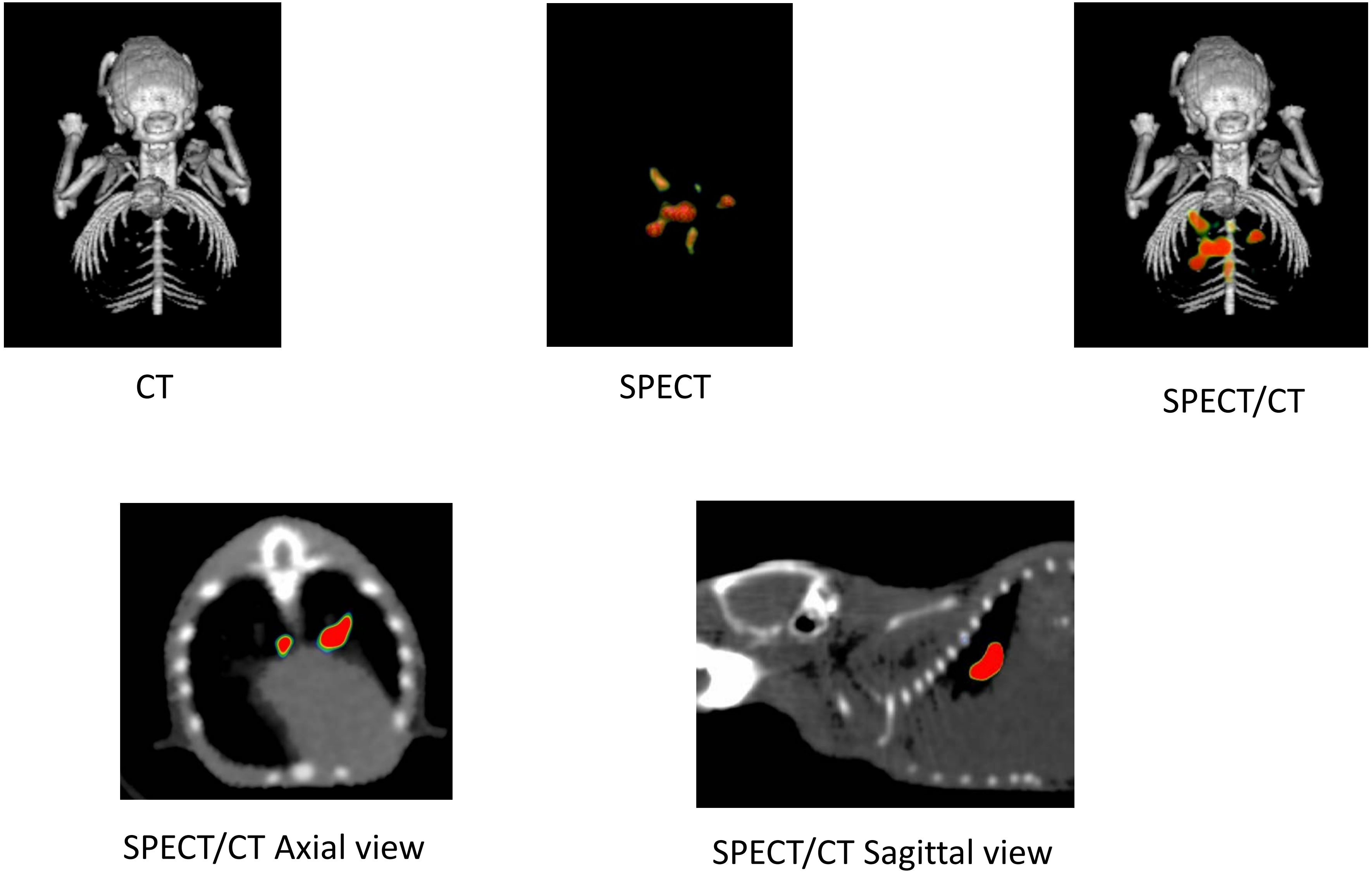

FIGURE S19. CT, SPECT, SPECT/CT, Axial and Sagittal view images of ^{177}Lu-scFvD2B in mouse No.3 bearing LNCaP micro-pulmonary tumors, collected at 72 h p.i.

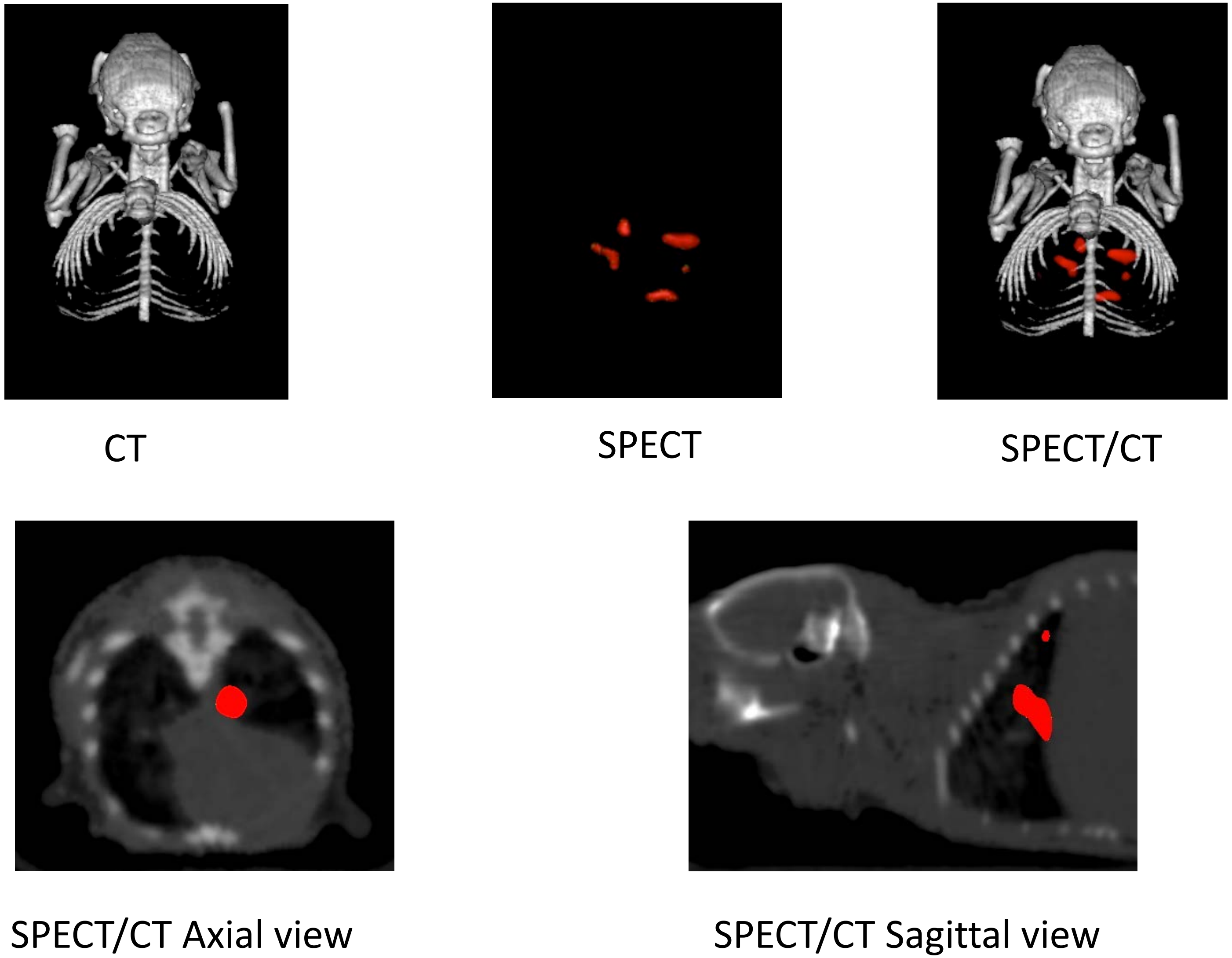

FIGURE S20. CT, SPECT, SPECT/CT, Axial and Sagittal view images of ^{177}Lu-scFvD2B in mouse No.3 bearing LNCaP micro-pulmonary tumors, collected at 192 h p.i.

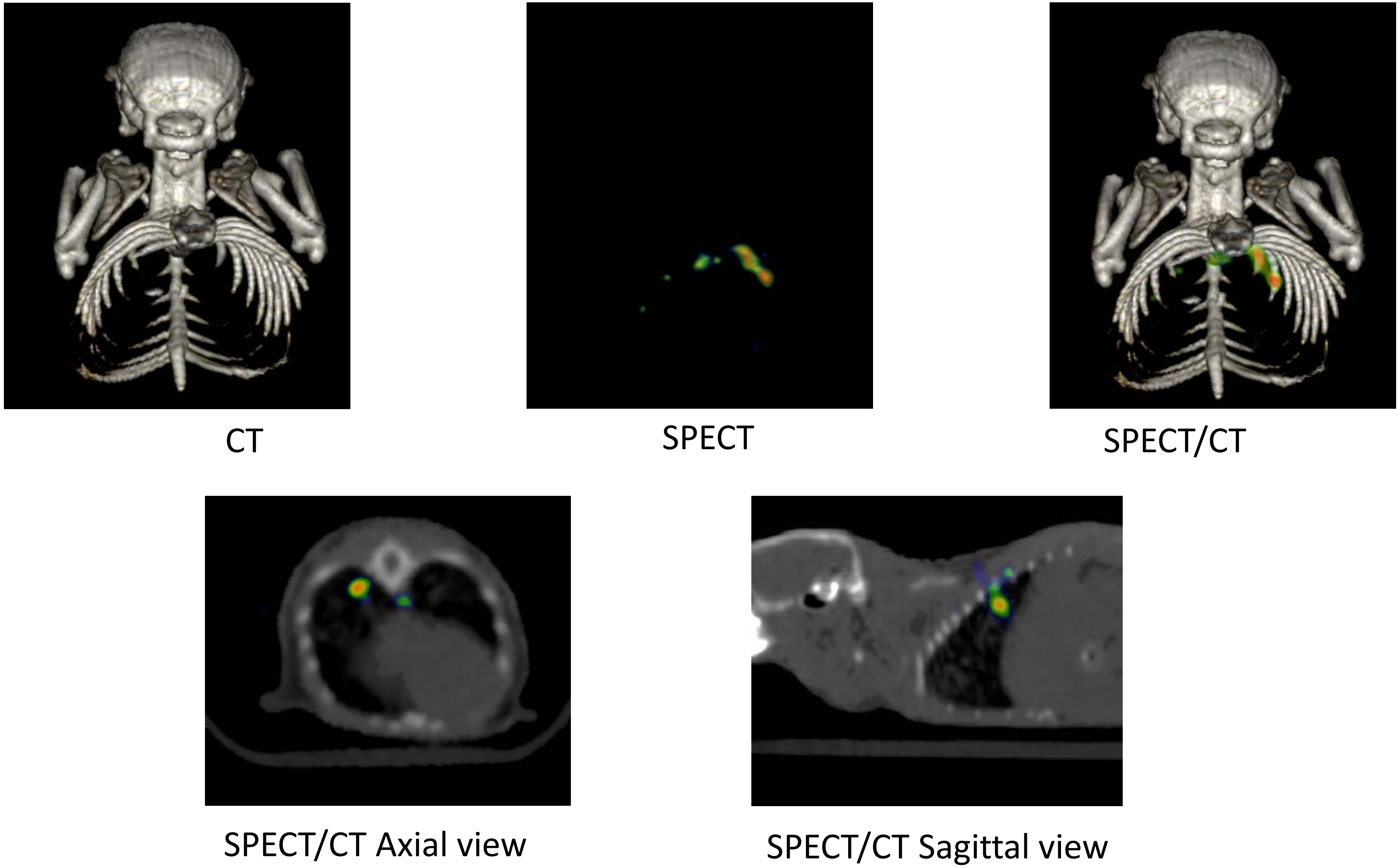

FIGURE S21. CT, SPECT, SPECT/CT, Axial and Sagittal view images of ^{177}Lu-iPSMA in mouse No.1 bearing LNCaP micro-pulmonary tumors, collected at 6 h p.i.

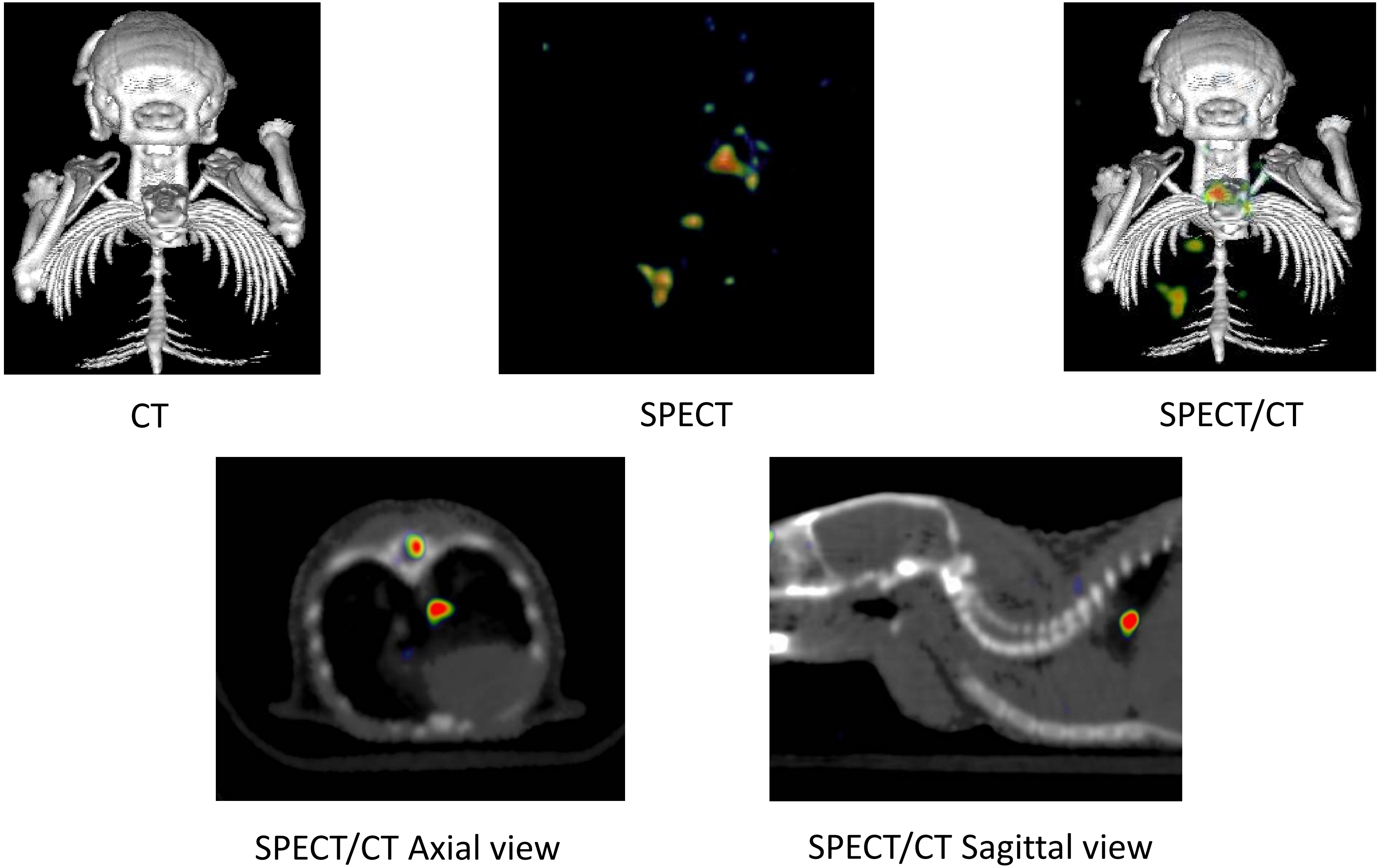

FIGURE S22. CT, SPECT, SPECT/CT, Axial and Sagittal view images of ^{177}Lu-iPSMA in mouse No.1 bearing LNCaP micro-pulmonary tumors, collected at 24 h p.i.

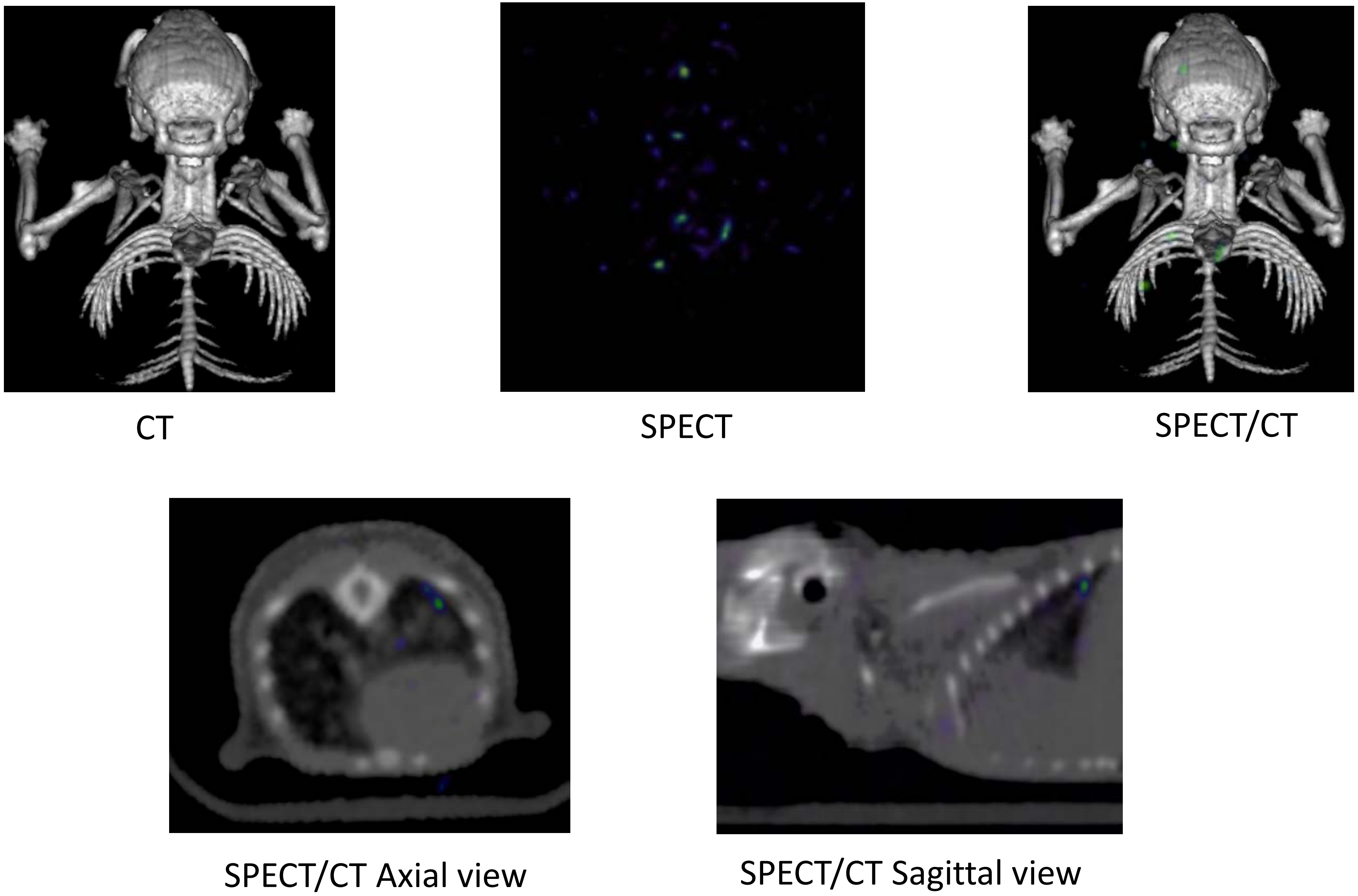

FIGURE S23. CT, SPECT, SPECT/CT, Axial and Sagittal view images of ^{177}Lu-iPSMA in mouse No.1 bearing LNCaP micro-pulmonary tumors, collected at 72 h p.i.

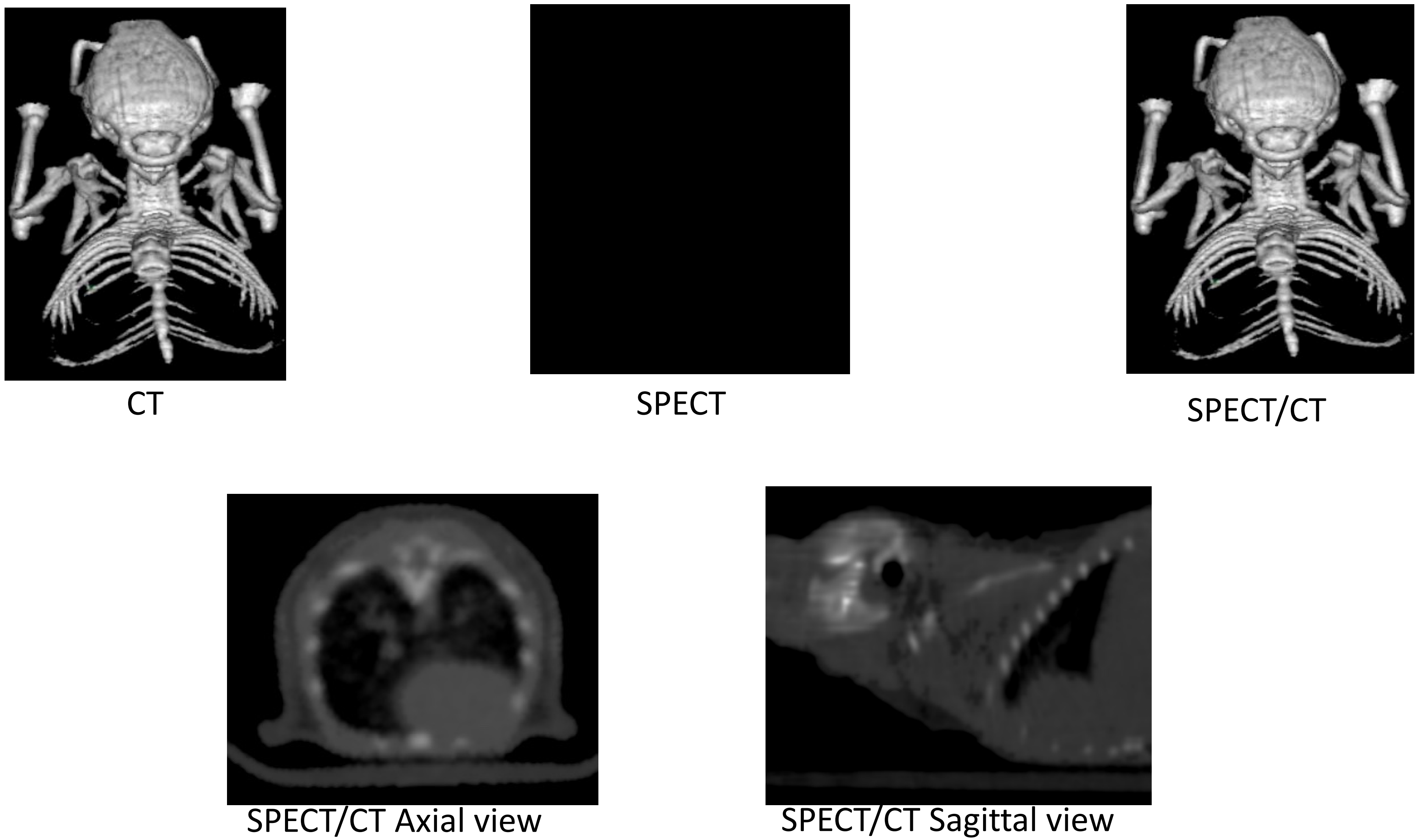

FIGURE S24. CT, SPECT, SPECT/CT, Axial and Sagittal view images of ^{177}Lu-iPSMA in mouse No.1 bearing LNCaP micro-pulmonary tumors, collected at 192 h p.i.

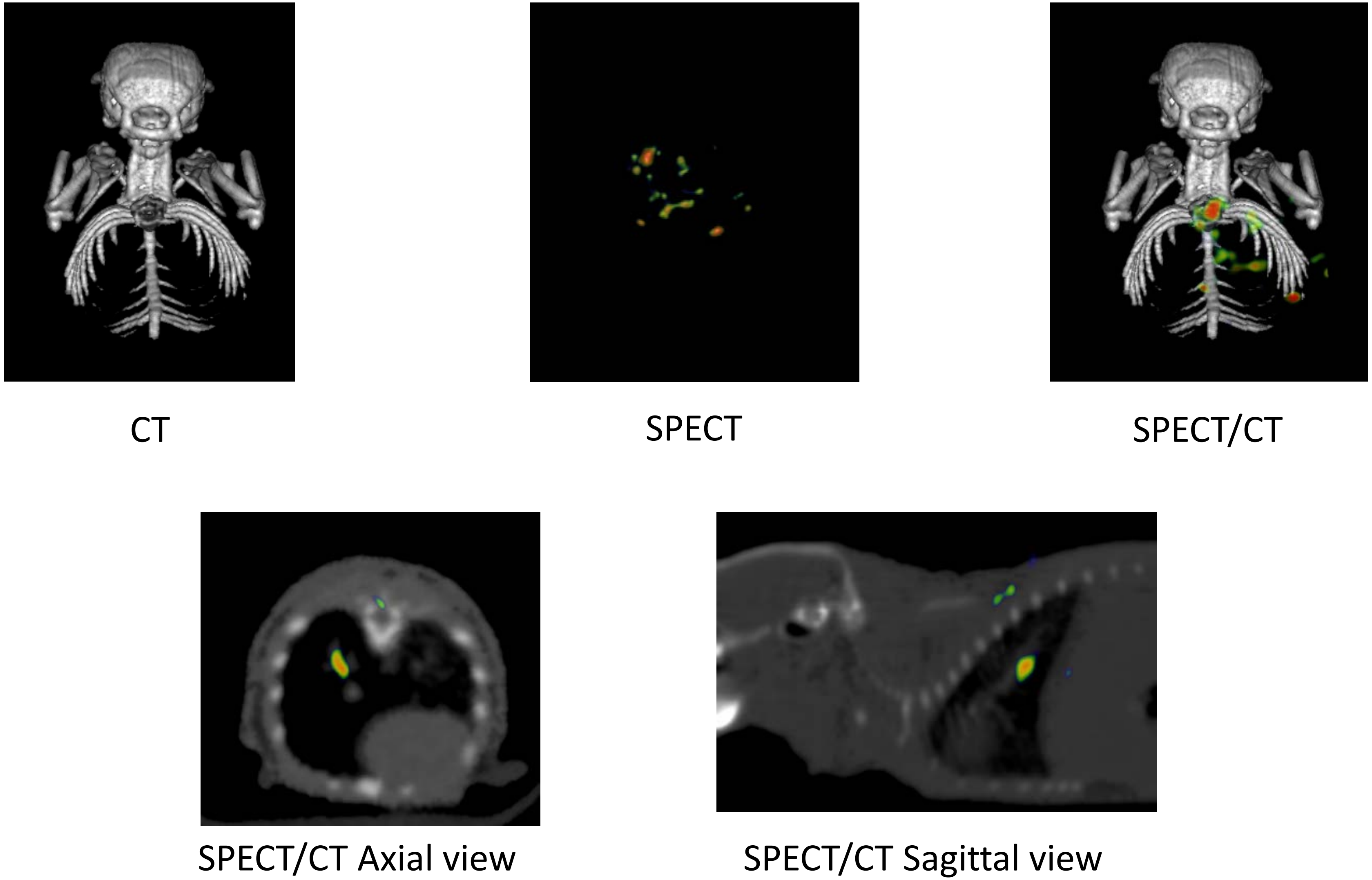

FIGURE S25. CT, SPECT, SPECT/CT, Axial and Sagittal view images of ^{177}Lu-iPSMA in mouse No.2 bearing LNCaP micro-pulmonary tumors, collected at 6 h p.i.

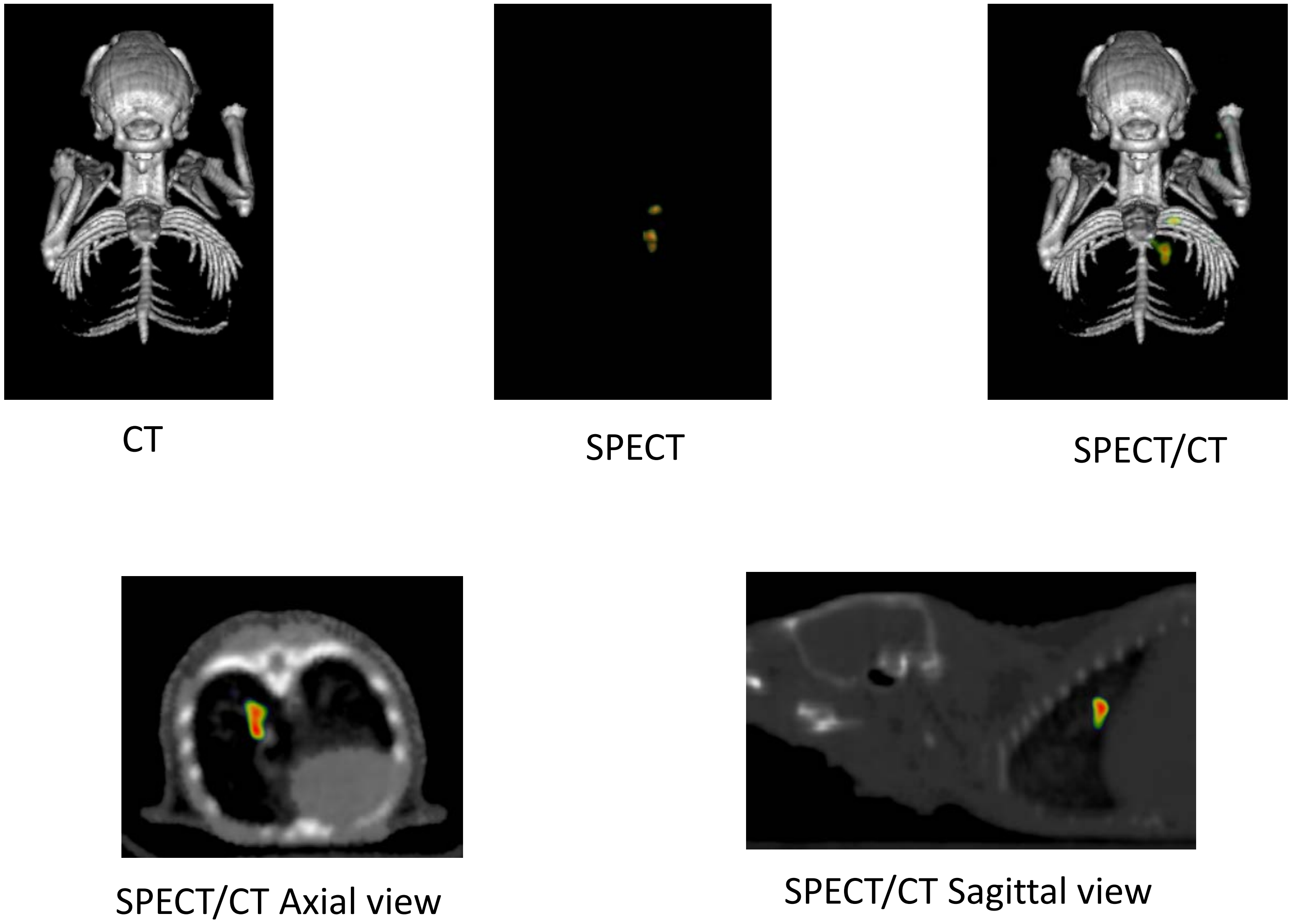

FIGURE S26. CT, SPECT, SPECT/CT, Axial and Sagittal view images of ^{177}Lu-iPSMA in mouse No.2 bearing LNCaP micro-pulmonary tumors, collected at 24 h p.i.

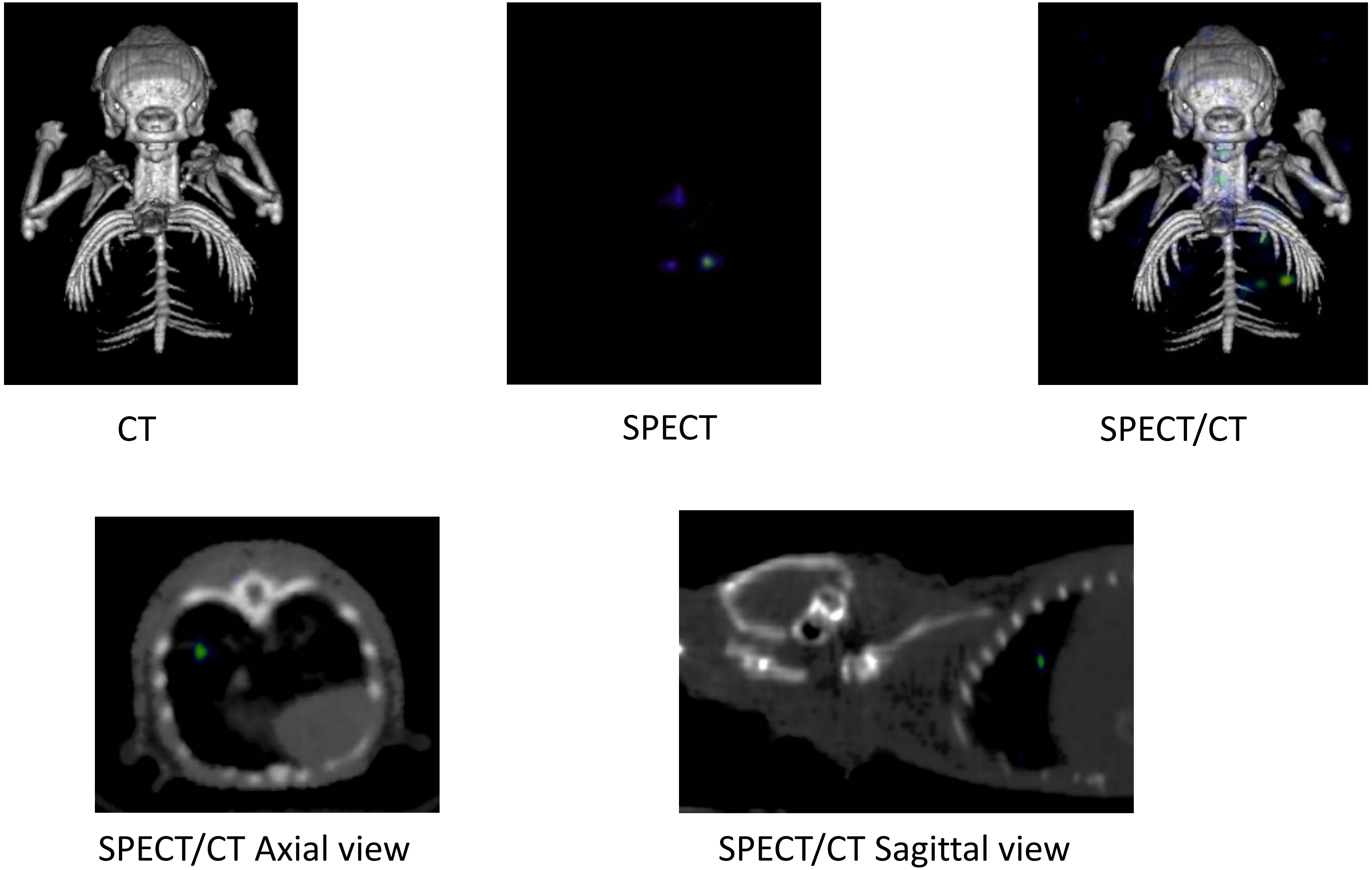

FIGURE S27. CT, SPECT, SPECT/CT, Axial and Sagittal view images of ^{177}Lu-iPSMA in mouse No.2 bearing LNCaP micro-pulmonary tumors, collected at 72 h p.i.

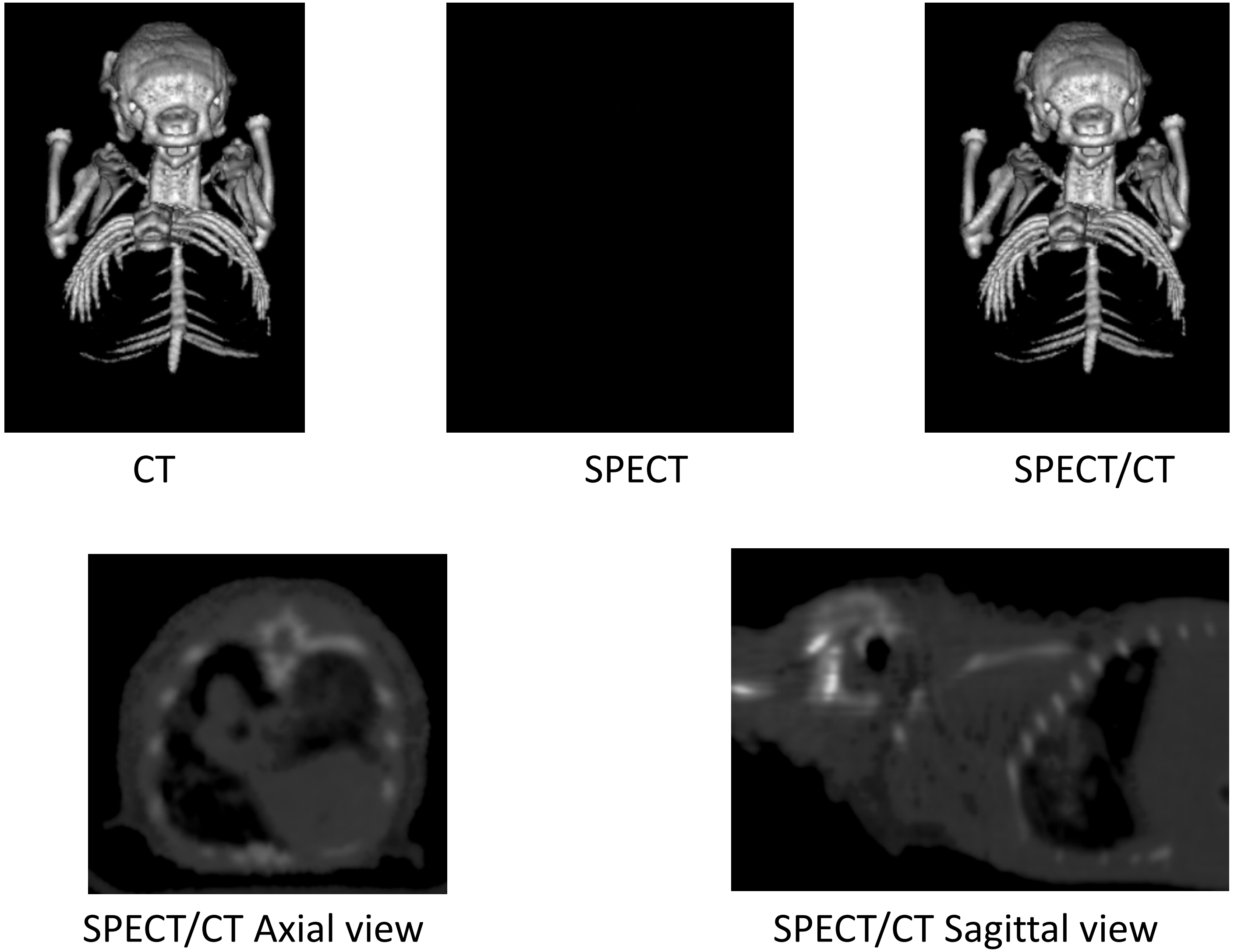

FIGURE S28. CT, SPECT, SPECT/CT, Axial and Sagittal view images of ^{177}Lu-iPSMA in mouse No.2 bearing LNCaP micro-pulmonary tumors, collected at 192 h p.i.

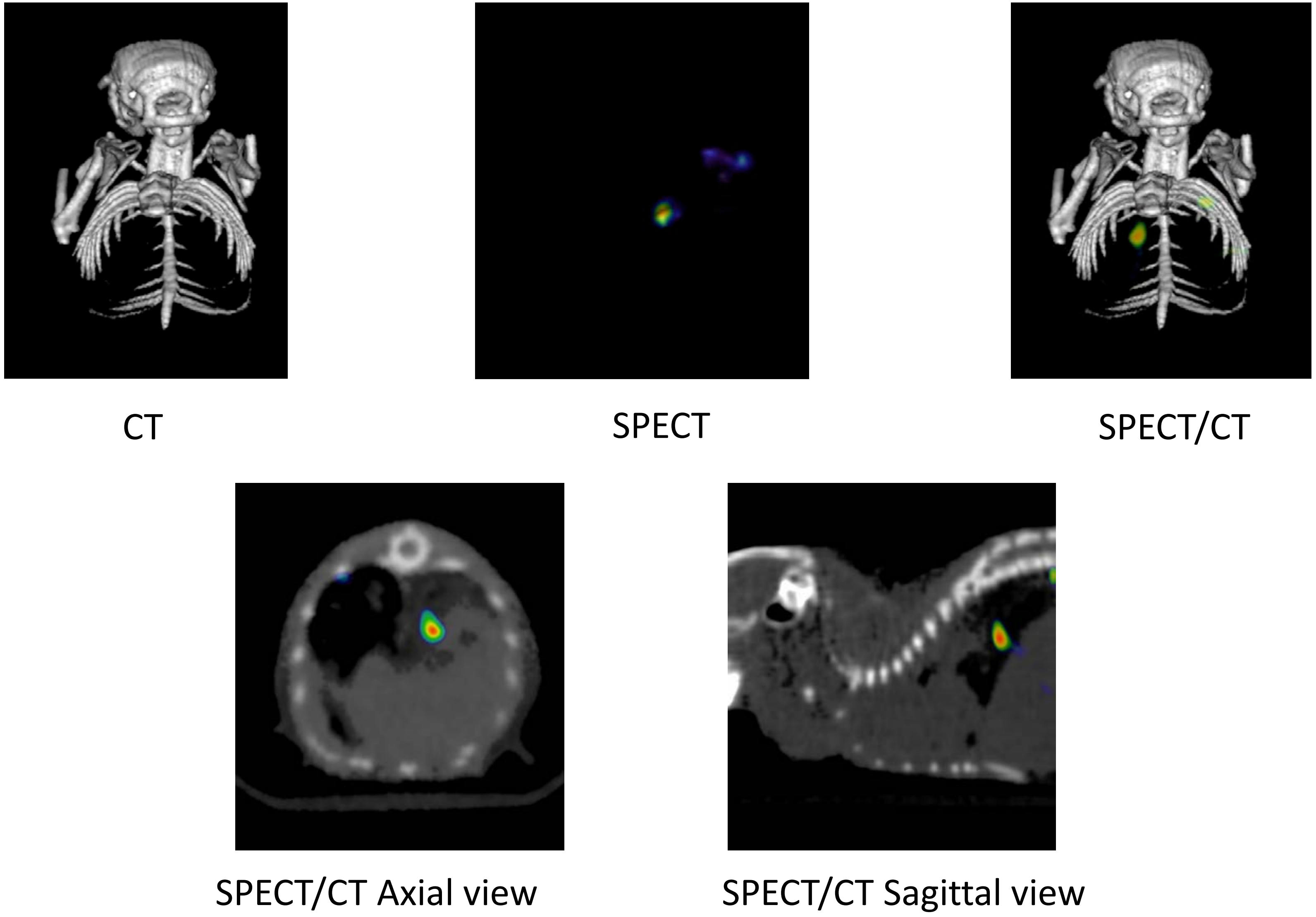

FIGURE S29. CT, SPECT, SPECT/CT, Axial and Sagittal view images of ^{177}Lu-iPSMA in mouse No.3 bearing LNCaP micro-pulmonary tumors, collected at 6 h p.i.

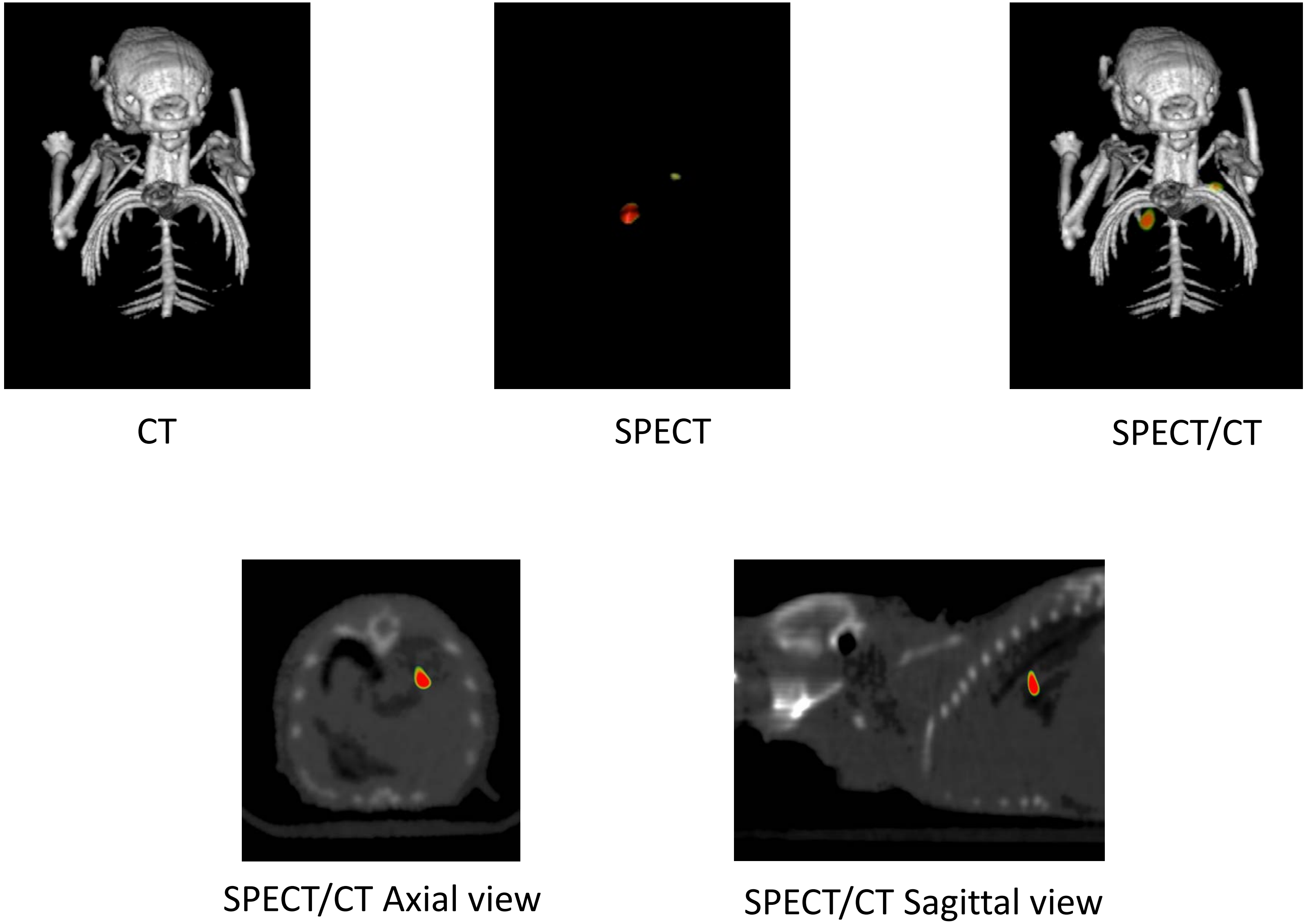

FIGURE S30. CT, SPECT, SPECT/CT, Axial and Sagittal view images of ^{177}Lu-iPSMA in mouse No.3 bearing LNCaP micro-pulmonary tumors, collected at 24 h p.i.

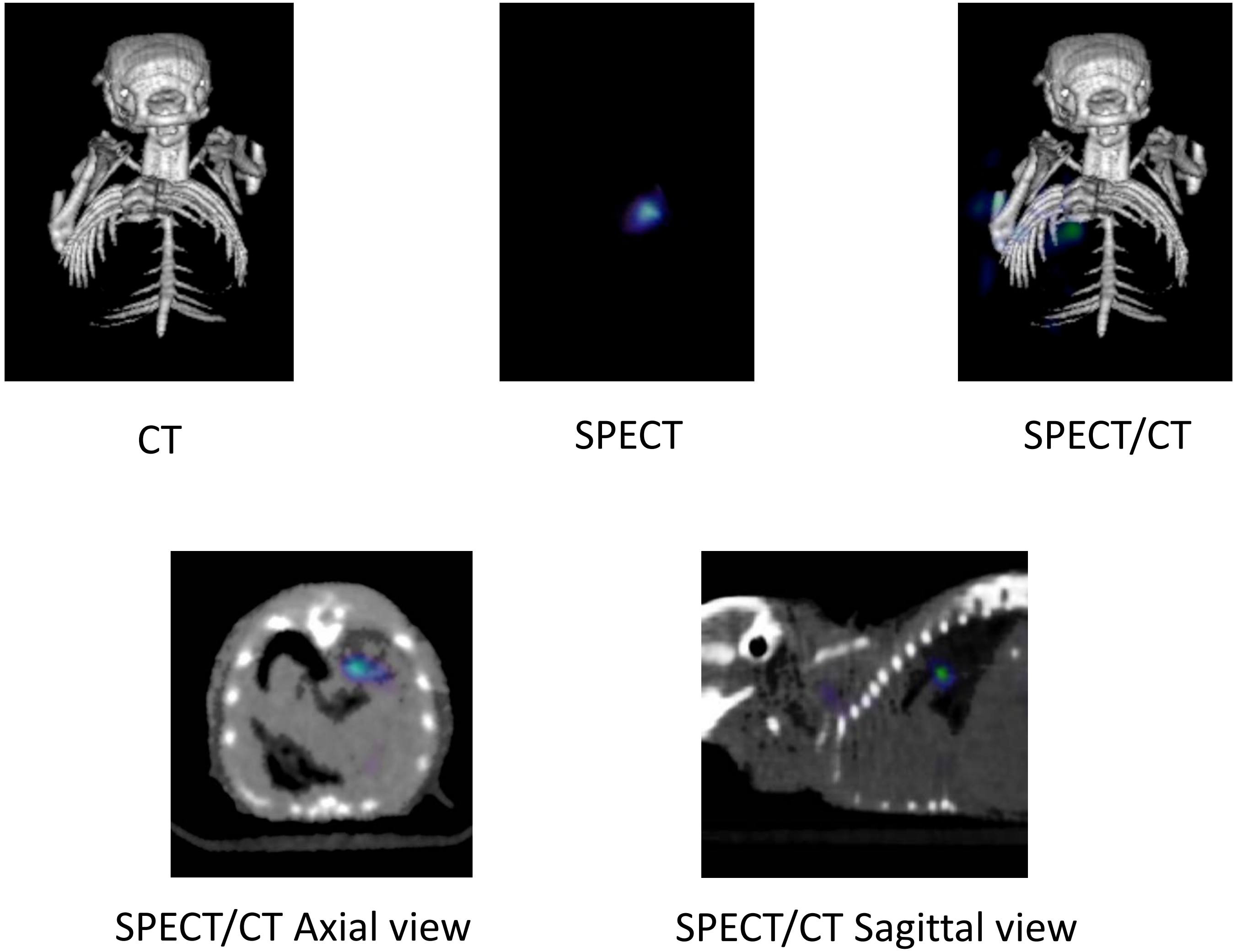

FIGURE S31. CT, SPECT, SPECT/CT, Axial and Sagittal view images of ^{177}Lu-iPSMA in mouse No.3 bearing LNCaP micro-pulmonary tumors, collected at 72 h p.i.

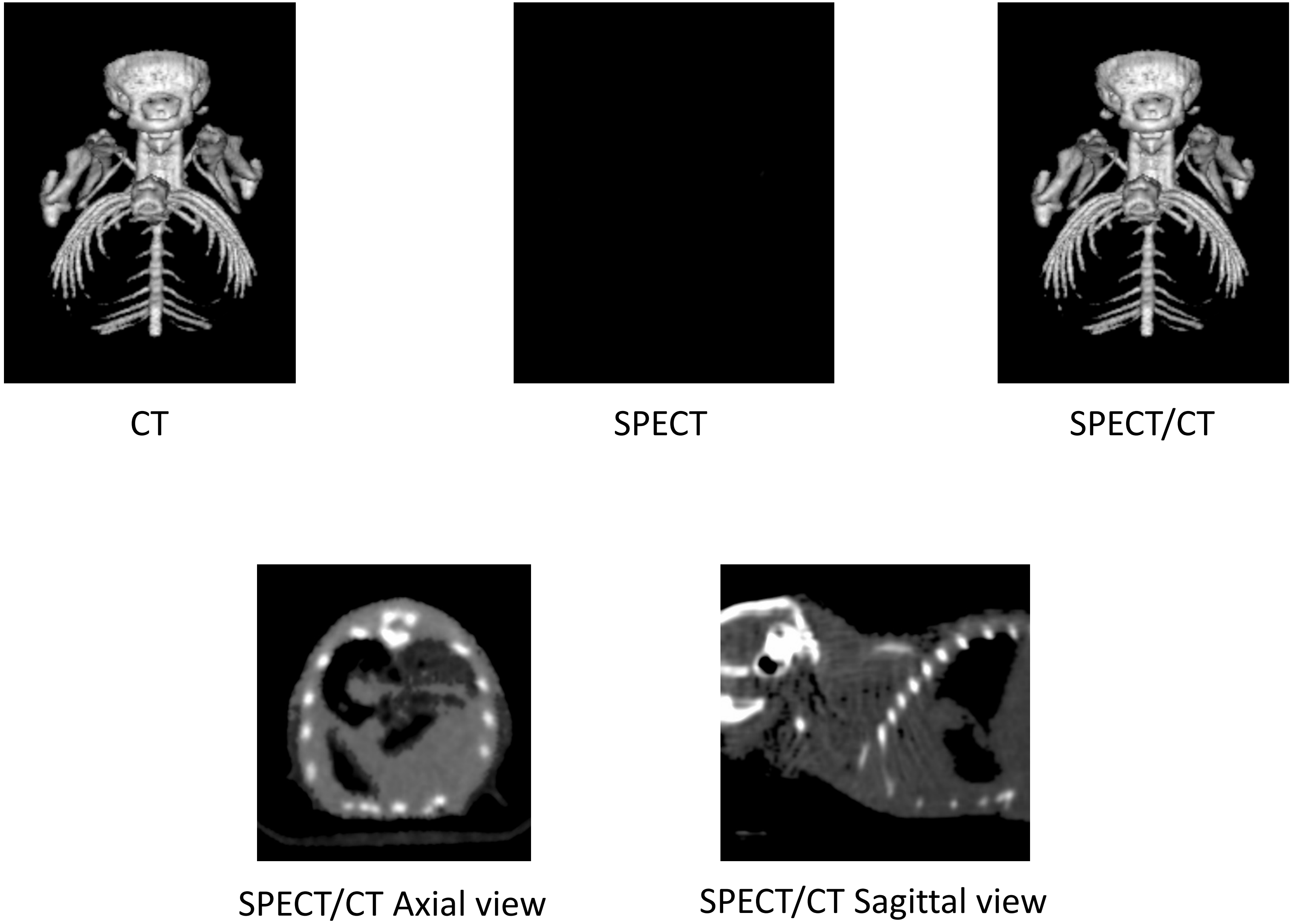

FIGURE S32. CT, SPECT, SPECT/CT, Axial and Sagittal view images of ^{177}Lu-iPSMA in mouse No.3 bearing LNCaP micro-pulmonary tumors, collected at 192 h p.i.
